# Red and processed meat consumption and the risk of pancreatic cancer: a systematic review and dose–response meta-analysis

**DOI:** 10.3389/fnut.2026.1829536

**Published:** 2026-07-14

**Authors:** Yina Lu, Minqi Gu, Yang Zhao, Liangze Ma, Yuchen Wei, Ziyang Liu, Yuying Wu, Xueru Fu, Jinliang Liang, Li Yang, Yaqin Su, Taifeng Chen, Dongdong Zhang, Fulan Hu, Dongsheng Hu, Ming Zhang

**Affiliations:** 1 Department of Biostatistics and Epidemiology, School of Public Health, Shenzhen University Medical School, Shenzhen, Guangdong, China; 2 Department of General Practice, The Affiliated Luohu Hospital of Shenzhen University Medical School, Shenzhen, China

**Keywords:** dose–response, meta-analysis, pancreatic cancer, processed meat, red meat

## Abstract

**Background:**

Previous studies suggest red and processed meat consumption may increase the risk of pancreatic cancer (PC); however, there is no consensus regarding the associations. A systematic review and meta-analysis were therefore performed to explore the relationship between red and processed meats and PC risk.

**Methods:**

We searched PubMed, EMBASE, and Web of Science for articles up to April 13, 2026, using random-effects models to calculate relative risks (RRs) and 95% CIs, restricted cubic splines for dose-response relationship, and subgroup/meta-regression for heterogeneity. This systematic review was prospectively registered on PROSPERO (registration number: CRD42022384026; https://www.crd.york.ac.uk/prospero/display_record.php?RecordID=384026).

**Results:**

Sixteen articles with 1,959,527 participants and 8,856 PC cases were included. There was a 16% increased risk of PC found in the highest versus lowest red meat consumption levels (RR = 1.16, 95% CI: 1.02–1.31). A linear association was observed between red (P non-linear = 0.656) and processed meats (P non-linear = 0.857) and PC risk, with a 10% increase in the risk of PC for each additional 100 g/d of red meat consumption (RR = 1.10, 95% CI: 1.00–1.21). For processed meat products, no significant association with PC risk was found.

**Conclusion:**

This finding indicates that increased red meat consumption may elevate the PC risk; therefore, daily red meat intake is recommended to be controlled. No evidence links processed meat to PC risk.

## 1 Introduction

Pancreatic cancer (PC) is experiencing a global increase in both incidence and mortality. In 2022, PC constituted approximately 3% of all cancer-related incidents globally and was the sixth leading cause of cancer-related mortality (1). PC is regarded as one of the most malignant diseases, with an unfavorable prognosis (2). The reduction of exposure to risk factors (such as obesity, smoking, and diet) is of paramount importance in the prevention of this disease which has become an increasingly serious public health concern (3, 4).

In recent years, red meat (including beef, pork, and lamb) and processed meat (including bacon, ham, hot dogs, and sausage) have accounted for a significant proportion of total dietary intake in many population groups, with global consumption increasing (5, 6). A wealth of research has established a clear connection between the consumption of red and processed meat and an increased likelihood of developing various health conditions, including cardiovascular disease, type 2 diabetes, and certain types of cancer (6–11). A meta-analysis conducted in 2012 and reviewing 11 prospective studies revealed that just an additional 50 grams (g) of processed meat consumed daily could lead to a 19% increase in PC risk; however, a similar positive association for red meat consumption was observed only in men (12). In a 2017 study, Zhao et al. (13) reported similar links between the highest and lowest levels of red and processed meat intake and PC risk, drawing on case–control research; yet a subsequent dose–response analysis showed no significant link between processed meat intake and the risk of PC (*p* = 0.90) (13). Notably, this dose–response meta-analysis included all articles referencing a particular sub-category of red or processed meat, such as pork, beef, lamb, bacon, and sausage (14, 15), which may suggest that the dosage results should be interpreted cautiously. In addition, the link between the consumption of red and processed meat and the risk of PC has garnered increasing attention from scholars, with a substantial number of high-quality studies published in recent years. The results of these studies have been inconsistent, however (16–19).

Presently, meta-analyses in nutritional epidemiology predominantly employ generic risk-of-bias instruments, including the Newcastle-Ottawa Scale; however, these tools may have limitations with regard to dietary assessment methodologies, baseline nutritional status, follow-up frequency, and between-group exposure differences maintained during the study, and therefore may not adequately evaluate the nutritional-specific methodological biases (13, 20). Research has demonstrated that Nutrition QUality Evaluation Strengthening Tools (NUQUEST), by integrating validated universal methodological standards with nutrition-specific assessment modules, can partially address these limitations (20). This approach enables a thorough evaluation of various components related to dietary exposure, including measurement validity, baseline exposure status, and maintenance of exposure differences during follow-up, leading to the inclusion of higher-quality studies. It is therefore necessary to adopt nutrition-focused risk of bias assessment tools to reduce the risk of bias and enhance the validity of conclusions, thereby enabling more rigorous and methodologically robust assessments of dose–response relationships.

Accordingly, we conducted a comprehensive systematic review and dose–response meta-analysis to examine the relationship between red meat and processed meat consumption and the risk of PC, using NUQUEST tool to rigorously evaluate risk of bias across included studies. We aimed to provide reliable evidence to inform the development of effective PC prevention policies and public health strategies.

## 2 Methods

This comprehensive review adhered to the guidelines set forth by the Preferred Reporting Items for Systematic Reviews and Meta-Analyses Protocols (PRISMA-P) (21), and was conducted according to the recent consensus guidelines for the design, conduct, and reporting of meta-analyses in biomedical research (22). It has been officially registered in the International Prospective Register of Systematic Reviews (PROSPERO) under the identifier CRD42022384026.

All deviations from the pre-registered protocol are transparently reported below. Although the PROSPERO-registered protocol intended to conduct subgroup analyses by specific types of red meat (e.g., beef, pork) and processed meat (e.g., ham, sausage, bacon), only a very limited number of included studies reported effect estimates for these individual meat subtypes. Due to the small number of available studies, such refined analyses were not performed, and we present overall estimates for total red meat and total processed meat consumption in relation to pancreatic cancer risk. For the assessment of methodological quality, the Newcastle–Ottawa Scale (NOS) was initially indicated in the PROSPERO registration; however, we used the NUQUEST scale in this review, as it is specifically developed for nutritional epidemiological studies of dietary exposures and is more suitable for evaluating studies investigating meat intake and cancer risk.

### 2.1 Selection criteria

The study selection criteria were as follows: focused on generally healthy human adult participants at baseline. Studies were excluded if they were not conducted in humans or enrolled participants with prevalent pancreatic cancer at baseline. Also, it had to be a study with PC morbidity as the outcome; adjusted relative risk (RR), hazard ratios (HR), or odds ratios (OR), and 95% confidence intervals (95% CI) had to be provided; and the diagnosis of PC had to be consistent with histological features. Narrative reviews, systematic reviews, meta-analyses, case reports, and low-quality studies were not considered. Additionally, studies that presented only a subset of red or processed meats (such as pork or sausage) and did not provide a comprehensive analysis were excluded due to the potential to affect the accuracy of dose–response analysis. In the present study, red meat referred to all unprocessed and processed mammalian red meat, including beef, pork, lamb, and other domesticated mammalian muscle meats. Processed meat was defined as meat preserved by salting, curing, smoking, fermentation, or addition of preservatives, including all types of processed meat products reported in original studies, regardless of whether they were derived from red meat. Red meat and processed meat were treated as distinct exposures and analyzed separately. White meat data and any data regarding the impact of various cooking techniques on PC risk were excluded, while all studies had to be limited to those involving humans.

### 2.2 Search strategy and article quality assessment

Comprehensive literature searches were carried out in PubMed (RRID:SCR_004846), EMBASE (RRID:SCR_001650), and Web of Science (RRID:SCR_022706) databases (Supplementary Table S1) up to April 13, 2026. Keywords included the general names of meat (such as meat, red meat, and processed meat), nouns of various types of meat (such as beef, pork, mutton, and bacon), and nouns related to PC (pancreatic neoplasms and pancreatic cancer, for instance). The included studies were then manually classified by study design. The inclusions and exclusions of the study were independently determined by two researchers (M. G and Y. Z).

NUQUEST served as the quality assessment tool for the included studies (20). The quality of each article was evaluated by M. G. or Y. Z. then analyzed together. Each study was independently rated by the two reviewers across five domains: selection, comparability, ascertainment (of outcomes or exposure), nutrition-specific issues, and an overall study rating. Domain ratings were assigned as “good” (low risk of bias), “neutral” (moderate risk of bias), or “poor” (high risk of bias). The overall rating for each study was then determined by synthesizing these domain-specific ratings (20).

### 2.3 Data extraction

For each investigation, information was gathered regarding the surname of the lead author, year of publication, country of study location, study name, study type, participants’ gender and age (mean or range), sample size, cases, exposure assessment, average or median intake by consumption level category, variables adjusted in multivariate models, and ORs/RRs/HRs for each type of meat consumption, with maximum control of confounders. If there was any ambiguity or uncertainty in the data extraction and quality assessment process, we consulted with a third researcher (M. Z).

### 2.4 Statistical analysis

For all data in the original literature, the HRs were directly used as RRs. The incidence of PC was <10%, which allowed for the approximation of the ORs to the RRs (23). The overall effect size across all studies was determined by utilizing the most refined RRs and 95% CIs from the highest quartile in comparison to the lowest. In instances where a study reported the results for males and females separately, the effect sizes were combined using a fixed-effects model. When the highest category was accessible, it was assumed that the open interval was identical to the adjacent one (24). Conversely, it was presumed that the lowest boundary was equal to zero if the lowest category was open (25).

A random-effects model was employed to evaluate the pooled RR values linked to consuming red and processed meats and PC risk. When the study exposure was indicated as “portion” or “serving,” we standardized the serving sizes to 120g for red meat and 50g for processed meat (26). In conducting a dose–response analysis, we applied the suggested increments of 100g per day for red meat and 50g per day for processed meat (26). To investigate the association between the intake of red and processed meats and the risk of PC, we employed restricted cubic splines that featured three knots at the 25th, 50th, and 75th percentiles to discern both linear and nonlinear relationships (27). We aggregated study-specific dose–response RRs (95% CIs) utilizing DerSimonian and Laird random-effects models for linear associations (28).

Heterogeneity among studies was evaluated using Cochran’s Q test, where a *p* < 0.1 indicated statistically significant heterogeneity. Additionally, the *I^2^* statistic was used, with 50% or higher values indicating low heterogeneity (29). To pinpoint possible sources of this heterogeneity, subgroup and meta-regression analyses were conducted by region, gender, design, sample size, and adjustments (body mass index, alcohol drinking, physical activity, vegetable and fruit consumption, and energy intake). To ensure the robustness of the findings, a sensitivity analysis was also carried out by systematically omitting one study at a time. Further, publication bias was evaluated via funnel plots and Egger’s test, where a *p* value below 0.05 indicated the presence of substantial bias (30). If bias was detected, the trim-and-fill method was employed to make the necessary adjustments.

All statistical analyses were conducted using Stata 17.0 (Stata Corp, College Station, TX, RRID:SCR_012763). A *p*-value below 0.05 was deemed to be statistically significant.

## 3 Results

### 3.1 Study characteristics and quality scores

**Figure 1:**
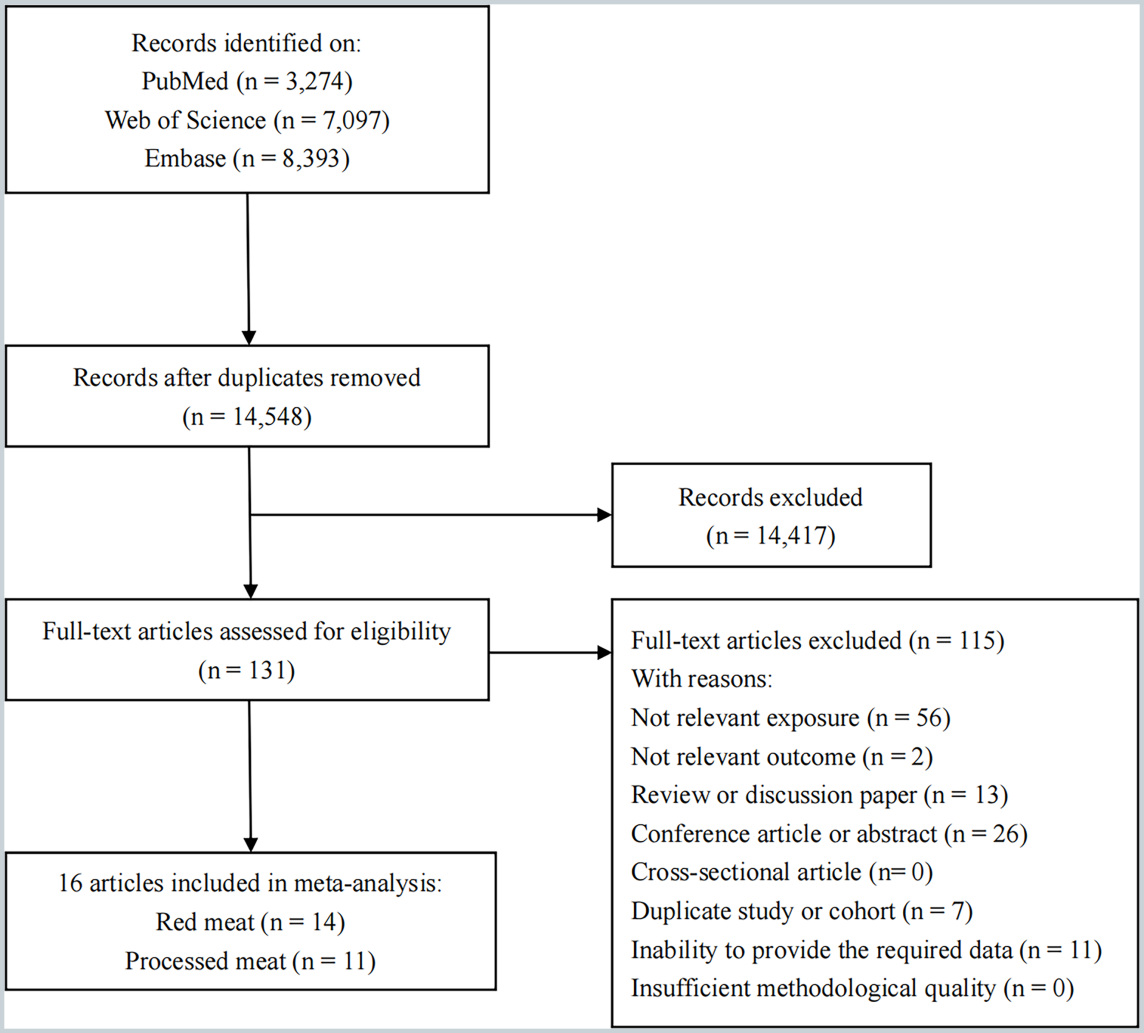
Flowchart of article selection.

Through a comprehensive search, 3,274 articles were sourced from PubMed, 8,393 from EMBASE, and 7,097 from Web of Science. Duplicate articles (4,216) and mismatched titles or abstracts (14,417) were excluded. After reviewing the complete texts, 115 articles were excluded, resulting in the inclusion of 16 pertinent studies. The flowchart detailing the article selection process can be found at Figure 1.

The sample size of eligible studies ranged from 509 (31) to 537,302 (32), with a total of 1,959,527 participants and 8,856 cases (8,137 red meat consumption cases, 5,686 processed meat consumption cases). Food frequency questionnaires assessed all meat exposures. Of the 14 red meat consumption studies (17, 19, 31–42), six were from countries in the European region (Italy, Finland, Sweden, Switzerland, and the United Kingdom), seven were from the United States, and one was from China. Of the 11 processed meat consumption studies (14, 17–19, 32, 35–39, 41), five were from countries in the European region (Italy, Finland, Sweden, and the United Kingdom), and six were from the United States. The main characteristics of the studies are shown in Table 1. The risk of bias for all 16 included studies was assessed using the NUQUEST. Fourteen studies were rated as neutral, indicating a moderate risk of bias, while two studies were classified as poor, reflecting a high risk of bias. Detailed ratings are presented in Supplementary Tables S2, S3.

**Table 1:** Characteristics of studies with PC risk included in the meta-analysis.

Study	Country	Design	Sample size	Age, years (mean or range)	Follow-up time	Gender	Cases	Exposure	Definition of red / processed meat	Intake levels of red and processed meat in the lowest and highest categories (g/day)	Repeat dietary assessment	Exposure assessment	Adjustment
Lyon et al. (31)	United States	Case control	509	40–79	NA	F/M	147	Red meat	Beef and pork	NR	NA	FFQ	Age, gender, smoking, alcohol
Ji et al. (33)	China	Case control	2,003	30–74	NA	F/M	451	Red meat	Pork chops, pork spareribs, pork feet, fresh pork (fat), fresh pork (lean), fresh pork (fat and lean), pork liver, other organ meats, beef and mutton	Lowest ≤13.7 (men) and 10.7 (women) servings/month; highest ≥37.8 (men) and 33 (women) servings/month	NA	FFQ	Age, income, smoking, green tea drinking, and response status
Tavani et al. (34)	Italy	Case control	8,352	≤ 75	NA	F/M	362	Red meat	Beef, veal and pork	2.34 portion for the low tertile of intake and 7.48 portion for the highest tertile	NA	FFQ	Age, gender, year of recruitment, education, smoking, alcohol, fat, vegetable and fruit consumption
Stolzenberg-Solomon (35)	Finland	Cohort	27,111	50–69	5 years	M	163	Red meat Processed meat	Beef, pork	Lowest ≤93.0 g/day; highest >175.6 g/day	Baseline only	FFQ-200	Age, smoking, energy intake
									Processed meats	Lowest ≤35.2 g/day; highest >100.6 g/day			
Michaud et al. (36)	United States	Cohort	88,802	30–55	18 years	F	178	Red meat	Beef, pork, lamb, processed meats, bacon, hot dogs, hamburger (excluding chicken)	Lowest 34 g/day; highest 167 g/day	4 times: 1980, 1984, 1986, 1990	FFQ-130	Smoking, height, BMI, PA, history of diabetes, caloric intake, and menopausal status
								Processed meat	Sausage, salami, and bologna	Lowest 0 serving; highest≥2 servings/week			
Larsson et al. (37)	Sweden	Cohort	36,616	53.75	18 years	F	172	Red meat	Minced meat (hamburgers, meatballs, meatloaf, etc); casserole with beef, pork or veal; and whole beef (steaks, roasts, etc).	Lowest <1.5 servings/week; highest >4.0 servings/week	2 times: 1987–1990, 1997	FFQ-350	Age, education, smoking, alcohol, BMI, energy intake, and energy-adjusted folate
								Processed meat	Sausage or hotdogs; bacon; ham, salami or lunch meat and blood pudding/sausage.	Lowest <1.5 servings/week; highest >4.0 servings/week			
Stolzenberg-Solomon (32)	United States	Cohort	537,302	50–71	5 years	F/M	836	Red meat	Bacon, beef (including that added to complex food mixtures, such as pizza, chili, lasagna, stew), cold cuts, ham, hamburger, regular hotdogs, liver, pork, sausage, and steak.	Lowest ≤19.0 (men)/≤13.0 (women) g/1000 kcal; highest >54.7 (men)/>43.7 (women) g/1000 kcal	2 times: baseline 1995–1996, 6-month later baseline	FFQ-124	Smoking, energy-adjusted saturated fat
								Processed meat	All types of cold cuts, bacon, ham, hotdogs, and sausages from red and white meats were included	Lowest ≤4.0 (men)/≤2.2 (women) g/1000 kcal; highest >18.4 (men)/>12.5 (women) g/1000 kcal			
Hu et al. (38)	Canada	Case control	24,771	20–76	NA	F/M	628	Red meat	Beef, pork, or lamb as a main dish; beef, pork, or lamb as a mixed dish (stew or casserole, pasta dish), and hamburger	Lowest ≤2 servings/week; highest ≥5.1 servings/week	NA	FFQ	Age, province, education, BMI, gender, alcohol, smoking, vegetable and fruit consumption, and energy intake
								Processed meat	Hot dogs, smoked meat, or corned beef; bacon and sausage	Lowest ≤0.94 servings/week; highest ≥5.42 servings/week			
Heinen et al. (39)	Netherlands	Cohort	120,852	55–69	13.3 years	F/M	350	Red meat	Beef, pork, minced meat (including beef and pork), liver, and other meat (e.g., horsemeat, lamb).	Lowest 45.8 (men)/36.2 (women) g/day; highest 145.9 (men)/130.4 (women) g/day	Baseline only	FFQ-150	Gender, age, energy intake, smoking, alcohol, history of diabetes, history of hypertension, BMI, vegetable and fruit consumption
								Processed meat	Meat items that had undergone some form of preservation (mostly treatment with nitrite salt, sometimes smoked or fermented).	Lowest 0 g/day; highest 35.7 (men)/25.6 (women) g/day			
Di Maso et al. (40)	Italy Switzerland	Case control	978	63	NA	F/M	326	Red meat	Beef, veal, pork, horsemeat, and half of the first course including meat sauce (e.g., lasagne, pasta/rice with bologna sauce)	Lowest <60 g/day; highest ≥90 g/day	NA	FFQ	Age, gender, study center, education, smoking, alcohol, BMI, vegetable and fruit consumption, and year of interview
Rohrmann et al. (41)	Europe	Cohort	477,202	51/52.7	10 years	F/M	865	Red meat	Beef, pork, mutton/lamb, horse and goat	Lowest 0 to <20 g/day; highest ≥80 g/day	Baseline only	FFQ	Education, height, weight, smoking, PA, history of diabetes and energy intake
								Processed meat	All meat products, including ham, bacon and sausages; small part of minced meat that has been bought as ready-to-eat product	Lowest 0 to <10 g/day; highest ≥40 g/day			
McCullough et al. (17)	United States	Cohort	138,266	≥ 60	15.7 years	F/M	1,156	Red meat	Hamburgers/ground beef, steak/roast, beef stew or pot pie, liver, and pork	Lowest ≤1.4 (men)/≤0.9 (women) servings/week; highest >5.2 (men)/>3.8 (women) servings/week	2 times: 1982, 1992	FFQ	Age, gender, smoking, BMI, history of diabetes, alcohol, saturated fat, and energy intake
								Processed meat	Hot dogs, lunch meats, sausage, and bacon	Lowest ≤0.5 (men)/≤0.1 (women) servings/week; highest >4.2 (men)/>2.2 (women) servings/week			
Rosato et al. (18)	Italy	Case control	2,177	58/61	NA	F/M	688	Processed meat	Ham, salami, bacon, sausages, and hot dogs	Lowest <10 g/day; highest ≥20 g/day	NA	FFQ-78	Age, gender, study center, year of interview, education, smoking, alcohol, BMI, vegetables and fruit consumption, and energy intake
Petrick et al. (19)	United States	Cohort	52,706	21–69	13 years	F	168	Red meat	Processed and unprocessed red meat—beef and pork	Lowest 0–16.80 g/day; highest 55.84–516.59 g/day	2 times: 1995, 2001; cumulative average	FFQ-68	Age, smoking, and energy intake
								Processed meat	Bacon, sausage, hot dogs, and lunchmeats	Lowest ~2.1 g/day; highest ~23.9 g/day			
Huang et al. (42) ^†^	United States	Cohort	184,542	45–75	17.5 years	F/M	1,618	Red meat	Beef, veal, lamb, pork, organ meats, ham, bacon, sausage, luncheon meat and corned beef	Lowest 0.0–14.1; highest 35.2–216.5 g/1000 kcal/day	Baseline only	FFQ	Age, ethnicity, gender, BMI, smoking, history of diabetes, family history of pancreatic cancer, and log-transformed total calories
Huang et al. (42)^‡^	United States	Cohort	66,793	51.9	10.6 years	F/W	266	Red meat	Fried beef, hamburger, ground beef, beef mixed dishes, pork/ham, lunch meat, hot dogs/sausages, and bacon	Lowest 0.0–26.3; highest 64.5–428.7 g/1000 kcal/day	Baseline only	FFQ	Age, ethnicity, gender, BMI, smoking, history of diabetes, family history of pancreatic cancer, and log-transformed total calories

F, female; M, male; FFQ, food-frequency questionnaire; BMI, body mass index; PA, physical activity; NR, not reported. ^†^Huang et al. (42): Multiethnic Cohort Study (MEC); ^‡^Huang et al. (42): Southern Community Cohort Study (SCCS).

### 3.2 High versus low red meat and processed meat consumption and PC risk

**Figure 2:**
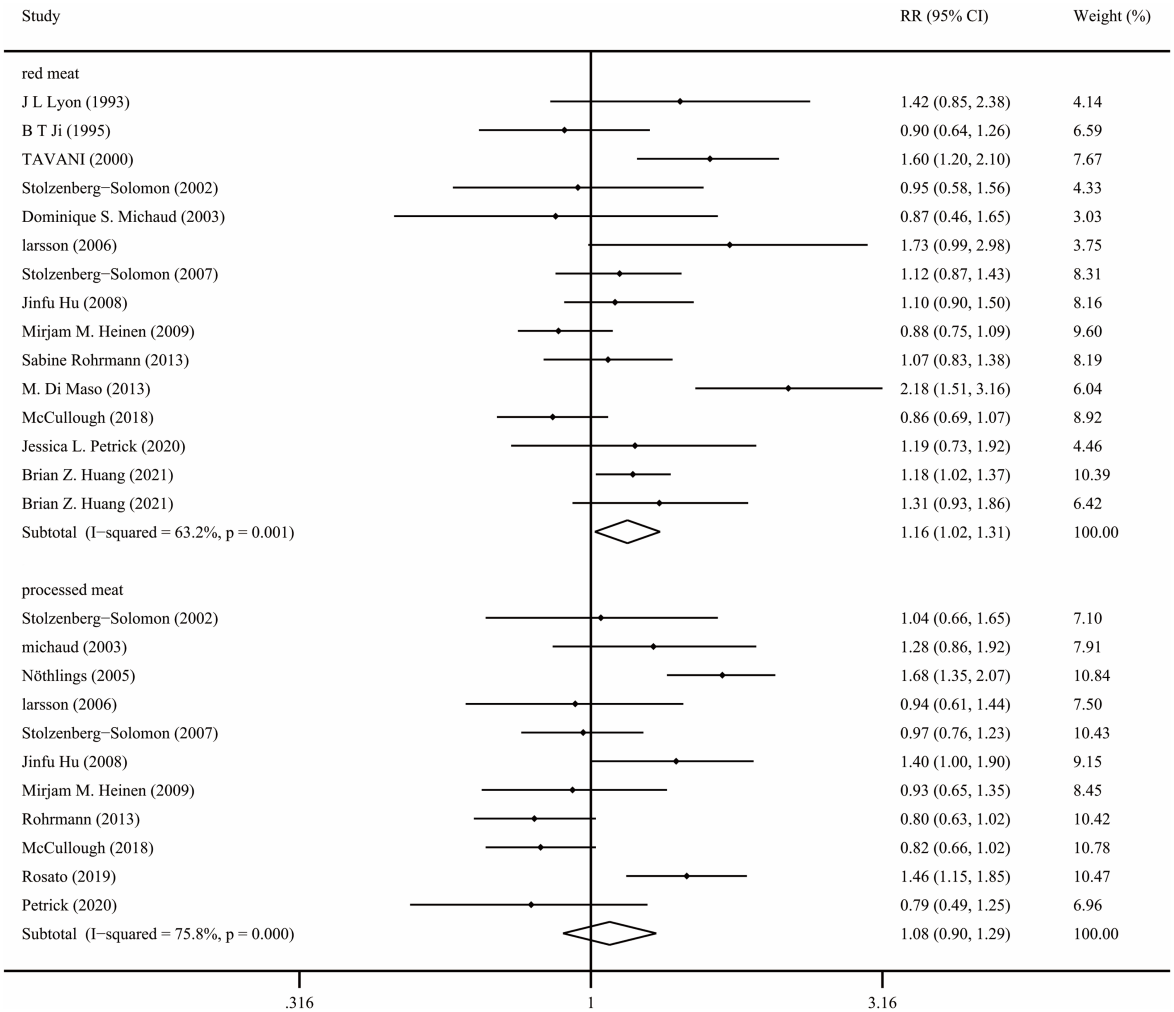
Forest plot of relative risk for red meat and processed meat consumption (highest versus lowest category) and pancreatic cancer risk.

The analysis of red meat involved 10 cohort studies and five case–control studies. A significant association was identified between the consumption of red meat and the risk of PC. The pooled RR was 1.16 (95% CI: 1.02–1.31; *I*^2^ = 63.2%, *P*_heterogeneity_ = 0.001, Figure 2). Additionally, Egger’s test did not reveal any signs of publication bias (*p* = 0.302; Supplementary Figure S1).

In examining the relationship between processed meat and the risk of PC, nine cohort studies and two case–control studies were analyzed. The overall effect size for processed meat demonstrated no significant association with the risk of PC (RR = 1.08, 95% CI: 0.90–1.29, *I*^2^ = 75.8%, *P*_heterogeneity_ < 0.001, Figure 2). Likewise, Egger’s test indicated an absence of publication bias (*p* = 0.302; Supplementary Figure S2).

### 3.3 Dose–response association between red and processed meat and PC risk

**Figure 3:**
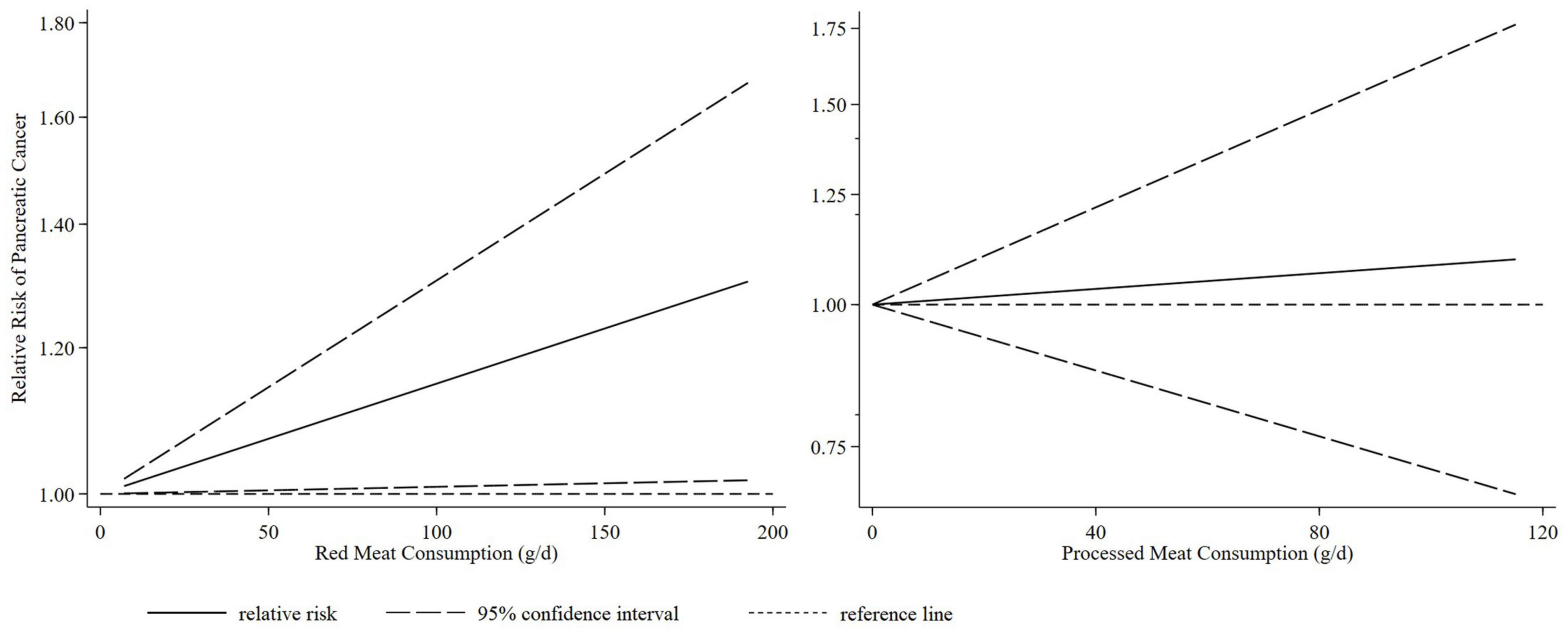
Dose–response association of meat consumption with risk of pancreatic cancer by restricted cubic splines.

**Figure 4:**
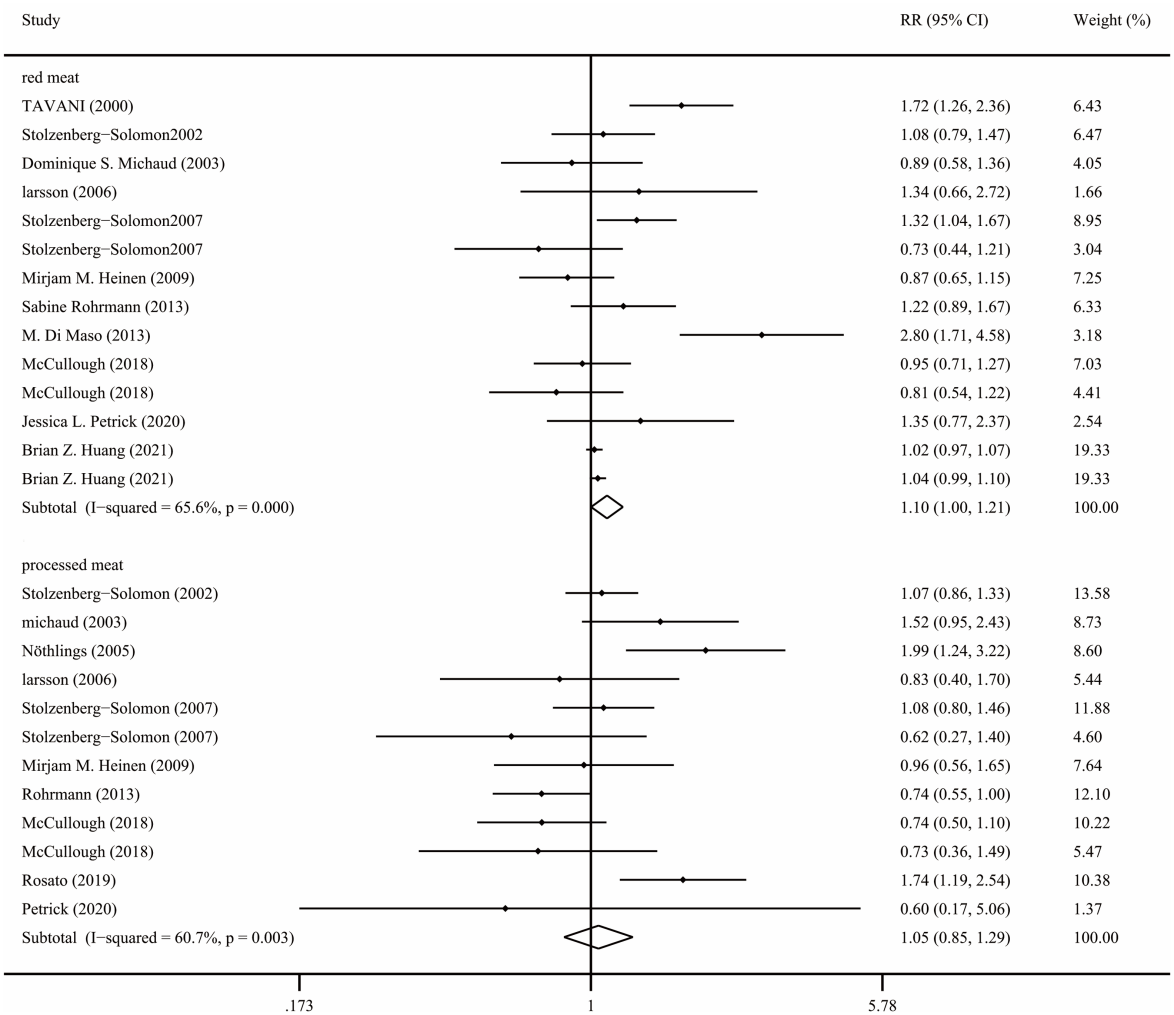
Forest plot of relative risk for pancreatic cancer risk per 100g/d and 50g/d increase in red meat and processed meat consumption.

A linear dose–response association was identified between red meat consumption and the risk of PC (*P*_non-linear_ = 0.656; Figure 3). The combined risk of developing PC rose by 10% (RR = 1.10, 95% CI: 1.00–1.21, *I*^2^ = 65.6%, *P*_heterogeneity_ < 0.001, Figure 4) for each additional 100 g/d of red meat consumption. There was no indication of publication bias detected (Egger’s test, *p* = 0.266; Supplementary Figure S3).

A linear relationship was observed between processed meat consumption and the risk of PC (*P*_non-linear_ = 0.857; Figure 3). When processed meat intake increased by 50 g/d, the combined RR (95% CI) for PC risk was 1.05 (0.85–1.29), with high heterogeneity (*I*^2^ = 60.7%, *P*_heterogeneity_ = 0.003, Figure 4). The Egger’s test indicated no publication bias (*p* = 0.769; Supplementary Figure S4).

### 3.4 Subgroup, meta-regression, and sensitive analyses

Meta-regression analysis indicated that region (*p* = 0.044) and sex (*p* = 0.035) might be potential sources of heterogeneity for the association between high versus low red meat consumption and PC risk. In addition, subgroup analysis by sex revealed a notable reduction in heterogeneity within both male (*I*^2^ = 0.0%, *P*_heterogeneity_ = 0.862) and female (*I*^2^ = 23.5%, *P*_heterogeneity_ = 0.271) subgroups, suggesting that sex may be an important source of heterogeneity. The same source of heterogeneity was observed in the association between red meat dose–response risk and PC risk, with *I*^2^ decreasing to 1.2% in the male subgroup (*p* = 0.314) and to 12.9% in the female subgroup (*p* = 0.328), respectively (Table 2).

**Table 2:** Subgroup and meta-regression analyses of relative risk of PC risk with red meat consumption.

Subgroup	High versus low category					Per 100g/d increment				
	*N*	RR (95%CI)	*I*^2^ (%)	*P^a^*	*P^b^*	*N*	RR (95%CI)	*I*^2^ (%)	*P^a^*	*P^b^*
All	15	1.16 (1.02–1.31)	63.2	0.001		14	1.10 (1.00–1.21)	65.6	0.000	
Region					0.044					0.748
Europe	6	1.30 (0.96–1.76)	82.0	0.000		6	1.36 (0.99–1.85)	76.7	0.001	
North America	8	1.10 (0.99–1.23)	15.4	0.309		8	1.03 (0.98–1.09)	23.2	0.245	
China	1	0.90 (0.64–1.26)	–	–						
Gender					0.035					0.952
Female	3	1.24 (0.86–1.79)	23.5	0.271		4	0.98 (0.74–1.31)	12.9	0.328	
Male	2	0.92 (0.69–1.21)	0.0	0.862		2	1.22 (1.01–1.48)	1.2	0.314	
Both	10	1.18 (1.02–1.38)	72.7	0.000		8	1.10 (0.98–1.23)	76.9	0.000	
Type of studies					0.189					0.896
Cohort	10	1.15 (0.97–1.36)	68.8	0.001		12	1.05 (0.96–1.16)	58.8	0.005	
Case–control	5	1.18 (0.96–1.44)	52.2	0.079		2	1.45 (1.03–2.04)	57.9	0.123	
Sample size					0.128					0.769
< 100,000	9	1.21 (1.03–1.41)	30.1	0.177		6	1.17 (0.96–1.43)	56.5	0.042	
≥ 100,000	6	1.12 (0.91–1.36)	79.3	0.000		8	1.08 (0.91–1.30)	72.8	0.001	
Adjustment										
BMI					0.933					0.931
Yes	8	1.16 (0.96–1.42)	75.4	0.000		10	1.05 (0.95–1.16)	65.1	0.002	
No	7	1.16 (0.99–1.36)	32.9	0.177		4	1.32 (1.05–1.64)	36.4	0.194	
Alcohol					0.201					0.983
Yes	6	1.38 (1.02–1.87)	80.3	0.000		5	1.36 (0.89–2.08)	81.9	0.000	
No	9	1.06 (0.96–1.17)	12.3	0.332		9	1.04 (0.99–1.09)	19.2	0.272	
Vegetable and fruit consumption					0.057					0.635
Yes	4	1.33 (0.91–1.94)	87.9	0.000		3	1.57 (0.83–2.98)	89.9	0.000	
No	11	1.09 (0.98–1.21)	20.6	0.247		11	1.04 (1.00–1.08)	6.4	0.383	
Energy intake					0.248					0.670
Yes	7	1.30 (1.02–1.66)	76.4	0.000		8	1.25 (0.95–1.65)	73.8	0.000	
No	8	1.06 (0.93–1.19)	29.9	0.189		6	1.03 (1.00–1.07)	0.0	0.658	

RR, relative risk, CI, confidence interval, BMI, body mass index. ^a^The P represents the heterogeneity observed within each subgroup, as determined by the Cochran Q test. ^b^The P is estimated by meta-regression.

Subgroup analysis also revealed reduced heterogeneity in the association between processed meat consumption and PC risk. Both the high versus low intake comparison and the dose–response analysis showed a clear decrease in heterogeneity within male and female subgroups, suggesting that sex is a consistent source of heterogeneity across different exposure assessments for processed meat and PC risk; however, the pooled results for both male and female subgroups remained statistically non-significant after stratification (Supplementary Table S4).

Additionally, in sensitivity analyses in which a single study was removed each time, no single study resulted in a change to the pooled effect size. The specific results are presented in the Supplementary Figures S5–S8.

## 4 Discussion

We believe that this meta-analysis study convincingly quantified the dose–response relationship between the consumption of red and processed meats and the risk of PC. It synthesized the latest evidence from case–control and cohort studies, suggesting additional red meat consumption significantly increased the risk of PC. A linear dose–response relationship was observed for red meat consumption in relation to PC risk. Specifically, each additional 100 g/day of red meat intake was associated with a 10% increased risk of PC (RR = 1.10, 95% CI: 1.00–1.21). Despite thorough investigation, no evidence emerged linking processed meat consumption to an elevated risk of PC. For processed meat, neither the highest versus lowest intake (RR = 1.08, 95% CI: 0.90–1.29) nor the per 50 g/day increment (RR = 1.05, 95% CI: 0.85–1.29) showed a statistically significant association with PC risk. However, given the wide confidence intervals and substantial heterogeneity (I^2^ = 75.8 and 60.7%, respectively), we cannot definitively exclude a potential weak or modest effect. Therefore, this finding should be interpreted with caution, particularly in the context of established evidence linking processed meat to other digestive tract cancer (43).

When examining high versus low consumption levels, this study revealed that elevated red meat intake was associated with an increased risk of PC, in alignment with previous research (12, 13). In contrast, there was no indication of a relationship between processed meat consumption and the risk of PC. We have incorporated more recently published articles than Zhao et al. (13) to provide new insights. Sun et al. (44) indicated an insignificant increase in the risk of PC linked to red and processed meat consumption; however, their study conducted a binary analysis of high versus low only. Considering the discrepancies in the grouping and dosage range across the various studies, a comprehensive dose–response analysis is essential for a more accurate interpretation of the findings.

Further dose–response analysis demonstrated a linear relationship between the intake of red and processed meats and PC risk in this meta. In contrast, Zhao et al. (13) observed that the risk of PC nonlinearly increased by 11% for 100 g/d red meat consumption in cohort studies. Although the magnitude of the risk increase for red meat in our analysis (10% per 100 g/d) is numerically close to that reported by Zhao et al., the nature of the dose–response relationship (a linear versus a nonlinear one) differs fundamentally. This divergence may be attributed to our study’s consideration of total consumption of red and processed meats, coupled with a more rigorous screening of high-quality articles which avoided the omission of the effects of other types of meat consumed on the same day when reporting sub-categories. Notably, most of the current studies are from developed countries in Europe and the United States. Given the differences in dietary customs and ethnic variations, future studies are expected to yield further high-quality data on the link between the consumption of red and processed meats and PC risk, thereby providing a more comprehensive evidence base for formulating PC prevention and control strategies.

Notably, the present study identified a significant but weak association between red meat intake and PC risk (RR = 1.16 for highest vs. lowest intake; RR = 1.10 per 100 g/day increment). These findings are biologically plausible in light of the well-established evidence linking red meat intake to colorectal cancer (CRC), a closely related digestive malignancy. A recent large-scale meta-analysis (43) confirmed that higher red meat intake was significantly associated with elevated risks of colon cancer (HR = 1.22, 95% CI 1.15–1.30), colorectal cancer (HR = 1.15, 95% CI 1.10–1.21), and rectal cancer (HR = 1.22, 95% CI 1.07–1.39). As the pancreas, colon, colorectum, and rectum are all key digestive organs exposed to metabolites from red meat digestion, the consistent evidence for CRC further supports the biological plausibility of the observed weak association between red meat intake and PC risk.

Consuming large amounts of red meat can lead to excessive absorption of heme iron, potentially triggering the development of cancer by heightening oxidative stress and causing DNA damage. This process may also facilitate the formation of harmful N-nitroso compounds (NOCs) (45, 46). Additionally, heterocyclic amines and polycyclic aromatic hydrocarbons present in red meat heated to high temperatures can compromise DNA integrity, further elevating the risk of cancer (47, 48). In addition, research demonstrated that red meat consumption could markedly elevate inflammatory cytokines levels, including C-reactive protein, interleukin-6, and tumor necrosis factor-*α* (49). The inflammatory factors may lead to the upregulation of cytidine deaminase, resulting in the increased instability of cancer-related genes, thereby enhancing their mutation and ultimately promoting carcinogenic processes (50). The above mechanisms align with our dose–response finding, which indicated a progressive, linear increase in PC risk with greater red meat intake. More red meat intake may lead to greater formation of heme iron, NOCs, genotoxic compounds, and inflammatory mediators, thereby producing a stronger carcinogenic effect, which potentially supports the dose–response relationship between red meat consumption and PC risk observed in our study. A physiological relationship exists between the risk of PC and the consumption of processed meat. The substantial quantities of nitrites and amides utilized in the manufacturing of processed meat products causes the body to produce NCOs which enter the bloodstream, causing cancer in the pancreas (51).

Our meta-regression indicated that geographic region was a source of heterogeneity in the association between red meat intake and PC risk. The divergence in risk estimates likely stems from regional differences in cooking practices; high-temperature methods like grilling and frying, more common in Western countries, generate NOCs that may elevate PC risk (51, 52). Further, a Western diet high in processed foods may synergistically increase PC risk through mechanisms like chronic inflammation (53), whereas Asian diets often include more vegetables, possibly attenuating this risk (54). Regional disparities in risk associations are influenced by additional factors, such as genetic variations in the metabolism of carcinogens (55). The risk associated with red meat intake is therefore not uniform; it is modified by population-specific culinary and dietary contexts.

Although significant between-gender heterogeneity was observed in the meta-regression analyses, the summary effect estimates for both men and women were not statistically significant. Therefore, the significant between-group heterogeneity identified in the high versus low meta-analysis may primarily reflect methodological heterogeneity across studies rather than a true biological difference. In contrast, the dose–response analysis for red meat and PC risk standardized exposure assessment by quantifying risk per 100 g/d increment, providing a more robust and comparable metric across studies. Men, lacking cyclical iron loss through menstruation, are more susceptible to iron overload from the heme iron in red meat (56, 57), which leads to sustained oxidative stress, DNA damage, and chronic inflammation (47, 48). Further, men tend to favor high-temperature cooking techniques, such as grilling and frying (58), which generate higher levels of known carcinogens, including heterocyclic amines (HCAs) and polycyclic aromatic hydrocarbons (PAHs) (52). In contrast, women often employ gentler methods like stewing or boiling, which produce significantly lower levels of these compounds. This behavioral difference in meat preparation could lead to higher exposure to dietary carcinogens among males.

The present study has several notable strengths. First, to our knowledge, this is the first meta-analysis exploring the association between red and processed meat and PC risk to apply the NUQUEST for quality assessment. It provided a nutrition-specific evaluation of dietary exposure, this specialized appraisal enhances methodological transparency and supports more robust interpretation of results within a nutritional epidemiologic framework. Although NUQUEST was applied as a rigorous nutrition-specific quality assessment tool, most included studies were rated as having neutral risk of bias, indicating moderate overall quality of the evidence base under this framework. This predominantly neutral rating was largely attributable to the strict, nutrition-specific criteria of the NUQUEST tool. Second, we uniformly converted the units of meat consumption across studies, employing the recommended serving size and increment units to ensure uniformity of data. Third, the analysis included the latest findings from both case–control and cohort studies. The rigorous screening process excluded low-quality studies and those only reporting a subset of meat types, resulting in more reliable and compelling conclusions. In addition, compared to previous researchers conducting meta-analyses, we employed more advanced dose–response associations and potential nonlinear trend modeling techniques to report specific associations between meat consumption and PC risk.

We also acknowledge certain limitations. First, studies incorporated into this meta were predominantly drawn from developed countries in the United States and Europe, and we acknowledge that the high-income countries in the study may have dietary habits, including higher intake of red meat and processed meat, that are different from other low-income and/or culturally diverse countries (59). Further research in low- and middle-income countries is thus proposed.

Second, the observed modest associations (RR = 1.16 for highest vs. lowest red meat intake; RR = 1.10 per 100 g/day increment) are inherently susceptible to residual confounding factors and measurement error, which is a well-recognized problem in observational epidemiology. To minimize this limitation as much as possible, we strictly prioritized and pooled only the most fully adjusted effect estimates from each included study, which had controlled for the core known confounders of PC risk, including age, sex, smoking status, BMI, diabetes, physical activity, ect. While we acknowledge that the unmeasured or incompletely adjusted factors, such as long-term dietary and specific lifestyle behaviors, may exert residual influence, the consistent use of maximally adjusted models across all studies substantially minimizes this risk and strengthens the robustness of our pooled findings.

Third, FFQs are the most widely applied instrument for large scale dietary assessment in nutritional epidemiologic research, especially in long-term prospective cohort studies. FFQs are subject to inherent limitations including measurement imprecision, recall bias, and misclassification of intake, which may distort or weaken the true dose–response relationship. To enhance comparability across studies, we standardized serving sizes to 120 g for red meat and 50 g for processed meat according to the World Cancer Research Fund global report (26). Even so, residual measurement error may still affect the precision of the estimated effect, warranting cautious interpretation of the dose–response findings.

Fourth, although we performed comprehensive subgroup analyses, meta-regression, and leave-one-out sensitivity analyses to explore potential sources of heterogeneity, these methods still could not fully explain the observed heterogeneity. Thus, random-effects model was used to provide a more conservative and robust summary effect. Additionally, pooling cohort and case–control studies may contribute to heterogeneity. However, meta-regression indicated that between-group heterogeneity by study design was not statistically significant. Pooling across designs was also necessary to ensure adequate statistical power, given the limited number of eligible studies available on this topic. Notably, case–control studies in nutritional epidemiology are susceptible to recall bias related to dietary exposure assessment, which may result in misclassification and represent an important limitation when interpreting pooled findings.

We restricted analyses to overall red meat and processed meat rather than specific subtypes (e.g., beef, pork, bacon) or cooking methods. Although this standardized exposure definition enhanced consistency across studies and supported reliable dose–response modeling, it may inadvertently omit biologically relevant variation in carcinogenic exposure.

The high heterogeneity observed in this study may be partly attributed to limitations in exposure assessment among the original included studies, such as the use of subjective FFQs with potential recall bias and inconsistent serving definitions. Future well-designed prospective studies are warranted to adopt gram-level precise quantification of meat intake and repeated exposure assessments during follow-up to better capture changes in dietary exposure and improve the reliability of findings.

## 5 Conclusion

In conclusion, this meta-analysis indicates that high consumption of red meat is associated with a moderately increased risk of pancreatic cancer. The linear dose–response association of our findings indicates that the risk of PC increased with rising consumption of red meat. These findings are consistent with previous studies on other malignancies such as colorectal cancer, all supporting the limitation of red meat intake, and align with existing public health policies and dietary guidelines, reinforcing the role of dietary and lifestyle modifications as an important strategy for cancer prevention. Although no positive effect was identified between processed meat consumption and PC risk, this result should not be interpreted lightly due to potential heterogeneity and limited evidence. Given the substantial global burden of pancreatic tumors, further well-designed research in low- and middle-income countries is proposed to address the current gap in knowledge regarding the relationship between red meat and processed meat consumption and the risk of PC.

## Data Availability

The original contributions presented in the study are included in the article/Supplementary material, further inquiries can be directed to the corresponding author.
