# Supplementary material for: Red and processed meat consumption and the risk of pancreatic cancer: a systematic review and dose–response meta-analysis
Source: Front Nutr. 2026 Jul 14;13:1829536. doi: 10.3389/fnut.2026.1829536 (PMC13407104; doi:10.3389/fnut.2026.1829536)
Supplement: Supplementary file 1 [file Table_1.DOCX]

**Supplementary materials:**

**Table S1**. Systematic literature review search terms and strategy.

**Table S2.** Assessment of quality of included cohort studies (NUtrition QUality Evaluation Strengthening Tool).

**Table S3.** Assessment of quality of included case control studies (NUtrition QUality Evaluation Strengthening Tool).

**Table S4.** Subgroup and meta-regression analyses of relative risk of PC risk with processed meat consumption

**Table S5.** Studies excluded after full-text review and reasons for exclusion

**Figure S1** Funnel plot of pancreatic cancer with high versus low red meat consumption.

**Figure S2.** Funnel plot of pancreatic cancer with high versus low processed meat consumption.

**Figure S3.** Funnel plot of pancreatic cancer with per 100g/d increment red meat consumption.

**Figure S4.** Funnel plot of pancreatic cancer with per 50g/d increment processed meat consumption.

**Figure S5.** Sensitivity analysis for pancreatic cancer with high versus low red meat consumption.

**Figure S6.** Sensitivity analysis for pancreatic cancer with high versus low processed meat consumption.

**Figure S7.** Sensitivity analysis for pancreatic cancer with per 100g/d increment red meat consumption.

**Figure S8.** Sensitivity analysis for pancreatic cancer with per 50g/d increment processed meat consumption.

**Supplementary references**.

**Table S1:** **Systematic literature review search terms and strategy.**

| **Search term for PubMed (n = 3,274)** |
| --- |
| #1 ("meat"[MeSH Terms] OR "Red meat"[MeSH Terms] OR "Pork meat"[MeSH Terms] OR "poultry"[MeSH Terms] OR "meat proteins"[MeSH Terms] OR "food"[MeSH Terms] OR "diet"[MeSH Terms] OR "sheep"[MeSH Terms] OR "goats"[MeSH Terms] OR "meat"[Title/Abstract] OR "Red meat"[Title/Abstract] OR "Pork meat"[Title/Abstract] OR "poultry"[Title/Abstract] OR "meat proteins"[Title/Abstract] OR "food"[Title/Abstract] OR "diet"[Title/Abstract] OR "sheep"[Title/Abstract] OR "goats"[Title/Abstract] OR "Meats"[Title/Abstract] OR "Meat Product"[Title/Abstract] OR "Product, Meat"[Title/Abstract] OR "Products, Meat"[Title/Abstract] OR "fresh meat"[Title/Abstract] OR "unprocessed meat"[Title/Abstract] OR "processed meat"[Title/Abstract] OR "further processed meat"[Title/Abstract] OR "animal protein"[Title/Abstract] OR "animal flesh"[Title/Abstract] OR "Red Meats"[Title/Abstract] OR "Beef"[Title/Abstract] OR "lamb"[Title/Abstract] OR "Lamb Meat"[Title/Abstract] OR "Lamb Meats"[Title/Abstract] OR "mutton"[Title/Abstract] OR "venison"[Title/Abstract] OR "Veal"[Title/Abstract] OR "fresh red meat"[Title/Abstract] OR "processed red meat"[Title/Abstract] OR "unprocessed red meat"[Title/Abstract] OR "Pork Meats"[Title/Abstract] OR "Pig Meat"[Title/Abstract] OR "Pig Meats"[Title/Abstract] OR "Pork"[Title/Abstract] OR "Bacon"[Title/Abstract] OR "Cured Ham"[Title/Abstract] OR "fresh poultry"[Title/Abstract] OR "processed poultry"[Title/Abstract] OR "iron"[Title/Abstract] OR "heme"[Title/Abstract] OR "heme iron"[Title/Abstract] OR "foods"[Title/Abstract] OR "diets"[Title/Abstract] OR "Ovis"[Title/Abstract] OR "Dall Sheep"[Title/Abstract] OR "Ovis dalli"[Title/Abstract] OR "Goat"[Title/Abstract] OR "Capra"[Title/Abstract] OR "Capras"[Title/Abstract]) |
| #2 ("pancreatic neoplasms"[MeSH Terms] OR "Pancreatic Cancer"[Title/Abstract] OR "Neoplasm, Pancreatic"[Title/Abstract] OR "Pancreatic Neoplasm"[Title/Abstract] OR "Neoplasms, Pancreatic"[Title/Abstract] OR "Pancreas Neoplasms"[Title/Abstract] OR "Neoplasm, Pancreas"[Title/Abstract] OR "Neoplasms, Pancreas"[Title/Abstract] OR "Pancreas Neoplasm"[Title/Abstract] OR "Cancer of Pancreas"[Title/Abstract] OR "Pancreas Cancers"[Title/Abstract] OR "Cancer of the Pancreas"[Title/Abstract] OR "Pancreas Cancer"[Title/Abstract] OR "Cancer, Pancreas"[Title/Abstract] OR "Cancers, Pancreas"[Title/Abstract] OR "Cancer, Pancreatic"[Title/Abstract] OR "Cancers, Pancreatic"[Title/Abstract] OR "Pancreatic Cancers"[Title/Abstract] OR "Pancreatic Carcinoma"[Title/Abstract] OR "Carcinoma, Pancreatic"[Title/Abstract] OR "Carcinomas, Pancreatic"[Title/Abstract] OR "Pancreatic Carcinomas"[Title/Abstract] OR "Pancreatic Acinar Carcinoma"[Title/Abstract] OR "Carcinoma, Pancreatic Acinar"[Title/Abstract] OR "Pancreatic Acinar Carcinomas"[Title/Abstract]) |
| #1 AND #2 |
| **Search term for Web of Science (n = 7,097)** |
| 1# TS=(meat OR "Red meat" OR "Pork meat" OR poultry OR "meat proteins" OR food OR diet OR sheep OR goats OR Meats OR "Meat Product" OR "Product, Meat" OR "Products, Meat" OR "fresh meat" OR "unprocessed meat" OR "processed meat" OR "further processed meat" OR "animal protein" OR "animal flesh" OR "Red Meats" OR Beef OR lamb OR "Lamb Meat" OR "Lamb Meats" OR mutton OR venison OR Veal OR "fresh red meat" OR "processed red meat" OR "unprocessed red meat" OR "Pork Meats" OR "Pig Meat" OR "Pig Meats" OR Pork OR Bacon OR "Cured Ham" OR "fresh poultry" OR "processed poultry" OR iron OR heme OR "heme iron" OR "foods" OR diets OR Ovis OR "Dall Sheep" OR "Ovis dalli" OR Goat OR Capra OR Capras) |
| 2# TS=( Pancreatic Neoplasms OR Pancreatic Cancer OR Neoplasm, Pancreatic OR Pancreatic Neoplasm OR Neoplasms, Pancreatic OR Pancreas Neoplasms OR Neoplasm, Pancreas OR Neoplasms, Pancreas OR Pancreas Neoplasm OR Cancer of Pancreas OR Pancreas Cancers OR Cancer of the Pancreas OR Pancreas Cancer OR Cancer, Pancreas OR Cancers, Pancreas OR Cancer, Pancreatic OR Cancers, Pancreatic OR Pancreatic Cancers OR Pancreatic Carcinoma OR Carcinoma, Pancreatic OR Carcinomas, Pancreatic OR Pancreatic Carcinomas OR Pancreatic Acinar Carcinoma OR Carcinoma, Pancreatic Acinar OR Pancreatic Acinar Carcinomas OR Pancreatic Neoplasm OR Pancreatic cancer, adult OR pancreatic adenomas OR pancreatic adenocarcinoma Pancreatic Diseases OR Disease, Pancreatic OR Diseases, Pancreatic OR Pancreatic Disease) |
| 1# AND 2# |
| **Search term for Embase (n=8,393)** |
| 1# Meat.mp. or processed meat/ or lamb meat/ or meat consumption/ or red meat/ or meat/ or Meats.mp. or Pork Meat.mp. or pork/ or Veal.mp. or veal/ or pork or Meats, Lamb.mp. or Meat, Lamb.mp. or Lamb Meats.mp. or lamb/ or Lamb Meat.mp. or lamb meat/ or beef/ or beef cattle/ or Beef.mp. or Red Meats.mp. or Meats, Red.mp. or Meat, Red.mp. or Red Meat.mp. or red meat/ or dietary.mp. or dietary intake/ or diets.mp. or diet/ or diet.mp. or mutton.mp. or mutton/ or sheep, Dall.mp. or Dall Sheep.mp. or sheep/ or Sheep.mp. or Ham, Cured.mp. or Cured Ham.mp. or Bacon.mp. or Pork.mp. or pork/ or Pig Meats.mp. or Meats, Pig.mp. or Meat, Pig.mp. or Pig Meat.mp. or Pork Meats.mp. or pig/ or Meats, Pork.mp. or Meat, Pork.mp. or hot dogs.mp. or sausage.mp. or Products, Meat.mp. or Product, Meat.mp. or Meat Product.mp. or Meat Products.mp. or foods.mp. or food/ or food.mp. |
| 2# Pancreatic Cancers.mp. or Cancers, Pancreatic.mp. or Cancer, Pancreatic.mp. or Pancreatic Cancer.mp. or Cancers, Pancreas.mp. or Cancer, Pancreas.mp. or Pancreas Cancer.mp. or Pancreas Cancers.mp. or pancreas adenocarcinoma/ or Cancer of Pancreas.mp. or Neoplasms, Pancreatic.mp. or Pancreas Neoplasm.mp. or Neoplasms, Pancreas.mp. or Neoplasm, Pancreas.mp. or Pancreas Neoplasms.mp. or Pancreatic Neoplasm.mp. or pancreas cancer/ or Neoplasm, Pancreatic.mp. or pancreas carcinoma/ or Pancreatic Neoplasms.mp. or pancreas tumor/ or pancreatic disease.mp. or pancreas disease/ |
| 1# AND 2# |

**Table S2. Assessment of quality of included cohort studies (NUtrition QUality Evaluation Strengthening Tool).**

| First author (year) | Selection of cohorts | | | | Rating | Comparability of cohorts | | | | Rating | Ascertainment of outcomes | | | | Rating | Nutrition-specific | | | | Rating | Overall |
| --- | --- | --- | --- | --- | --- | --- | --- | --- | --- | --- | --- | --- | --- | --- | --- | --- | --- | --- | --- | --- | --- |
|  | a | b | c | d |  | e | f | g | h |  | i | j | k | l |  | m | n | o | p |  |  |
| Stolzenberg-Solomon et al. (2002) | 2 | 3 | 3 | 2 | 0 | 2 | 4 | 3 | 1 | 0 | 1 | 2 | 4 | 1 | 0 | 2 | 1 | 3 | 1 | 0 | 0 |
| Michaud et al. (2003) | 1 | 3 | 1 | 3 | 0 | 3 | 3 | 1 | 1 | 0 | 1 | 2 | 2 | 1 | + | 2 | 1 | 3 | 1 | 0 | 0 |
| Nöthlings et al. (2005) | 2 | 4 | 1 | 3 | 0 | 3 | 4 | 3 | 1 | - | 1 | 2 | 2 | 1 | + | 3 | 1 | 3 | 1 | 0 | 0 |
| Larsson et al. (2006) | 1 | 3 | 1 | 2 | 0 | 2 | 3 | 1 | 1 | 0 | 1 | 2 | 4 | 1 | 0 | 3 | 1 | 3 | 1 | 0 | 0 |
| Stolzenberg-Solomon et al. (2007) | 2 | 4 | 2 | 3 | 0 | 3 | 3 | 2 | 2 | 0 | 1 | 3 | 3 | 1 | 0 | 3 | 1 | 3 | 2 | 0 | 0 |
| Heinen et al. (2009) | 1 | 3 | 1 | 2 | 0 | 2 | 3 | 1 | 1 | 0 | 1 | 2 | 2 | 1 | + | 3 | 2 | 3 | 1 | 0 | 0 |
| Rohrmann et al. (2013) | 1 | 3 | 1 | 2 | 0 | 2 | 4 | 1 | 1 | 0 | 1 | 2 | 3 | 1 | 0 | 2 | 2 | 3 | 1 | 0 | 0 |
| McCullough et al. (2017) | 1 | 3 | 1 | 3 | 0 | 2 | 4 | 1 | 1 | 0 | 1 | 2 | 3 | 1 | 0 | 3 | 1 | 3 | 1 | 0 | 0 |
| Petrick et al. (2020) | 1 | 4 | 1 | 2 | 0 | 2 | 4 | 1 | 1 | 0 | 1 | 1 | 3 | 1 | 0 | 2 | 1 | 3 | 1 | 0 | 0 |
| Huang et al. (2021) | 1 | 3 | 1 | 2 | 0 | 3 | 4 | 3 | 1 | - | 1 | 1 | 4 | 1 | 0 | 2 | 1 | 3 | 1 | 0 | 0 |

a：The groups being studied are selected from source populations that are comparable in all respects other than the exposures under investigation.

b：The study indicates how many of the people asked to take part did so in each of the groups being studied.

c：The likelihood that some eligible subjects might have the outcome at the time of enrolment is assessed and taken into account in the analysis.

d：The exposure assessment method is adequate to differentiate exposure among study groups.

e：The percentage of individuals or clusters recruited into each arm of the study that dropped out before the study was completed is reasonable.

f：Comparison is made between full participants and those lost to follow-up, by exposure.

g：Exposure level or prognostic factor is assessed more than once.

h：The main potential confounders are identified and taken into account in the design and/or analysis.

i：The outcomes are clearly defined.

j：The assessment of outcome is made blind to exposure. If the study is retrospective, this may not be applicable.

k：Where blinding was not possible, there is some recognition that knowledge of exposure could have influenced the assessment of outcome.

l：Evidence from other sources is used to demonstrate that the method of outcome assessment is valid and reliable.

m：The frequency and quantity of the exposure under study are accurately and reliably measured.

n：The relevant exposure at baseline is measured and taken into account.

o：The baseline exposure differences between the groups have been maintained over the course of the study.

p：The interval between the exposure and outcome is of sufficient duration to observe an effect, if there is one.

+：Good；0：Neutral ；-Poor.

1：YES; 2: PROBABLY YES; 3: PROBABLY NO; 4: NO

**Table S3. Assessment of quality of included case control studies (NUtrition QUality Evaluation Strengthening Tool).**

| First author (year) | Creation of study groups | | | | Rating | Comparability of study groups | | | | Rating | Exposure ascertainment | Rating | Nutrition-specific | | Rating | Overall |
| --- | --- | --- | --- | --- | --- | --- | --- | --- | --- | --- | --- | --- | --- | --- | --- | --- |
|  | a | b | c | d |  | e | f | g | h |  | i |  | j | k |  |  |
| Lyon et al. (1993) | 2 | 3 | 1 | 1 | 0 | 2 | 4 | 4 | 1 | - | 4 | - | 3 | 2 | 0 | - |
| Ji et al. (1995) | 1 | 2 | 1 | 2 | + | 1 | 2 | 4 | 1 | 0 | 3 | 0 | 3 | 1 | 0 | 0 |
| Tavani et al. (2000) | 2 | 2 | 1 | 1 | + | 1 | 3 | 4 | 1 | 0 | 2 | 0 | 3 | 2 | 0 | 0 |
| Hu et al. (2008) | 1 | 3 | 1 | 2 | 0 | 3 | 1 | 4 | 1 | 0 | 3 | 0 | 3 | 2 | 0 | 0 |
| DiMaso et al. (2013) | 2 | 3 | 1 | 1 | 0 | 3 | 3 | 4 | 1 | - | 3 | 0 | 3 | 2 | 0 | - |
| Rosato et al. (2018) | 1 | 2 | 1 | 1 | + | 1 | 1 | 4 | 1 | 0 | 2 | 0 | 3 | 1 | 0 | 0 |

a：The cases and controls are taken from comparable populations.

b：The same exclusion criteria are used for both cases and controls.

c：Cases are clearly defined and differentiated from controls.

d：It is clearly established that controls are non-cases.

e：The percentage of individuals in the study who participated as cases and controls is reasonable.

f：Measures will have been taken to prevent knowledge of primary exposures influencing case ascertainment.

g：Comparison is made between participants and non-participants to establish their similarities or differences.

h：The main potential confounders are identified and taken into account in the design and/or analysis.

i：The exposure under study is accurately and reliably measured.

j：The baseline exposure differences between the groups have been maintained over the course of the study.

k：The interval between the exposure and outcome is of sufficient duration to observe an effect, if there is one.

+：Good；0：Neutral ；-Poor.

1：YES; 2: PROBABLY YES; 3: PROBABLY NO; 4: NO

**Table S4. Subgroup and meta-regression analyses of relative risk of PC risk with processed meat consumption**

| **Subgroup** | **high versus low** | | | | | **per 50g/d increment** | | | | |  |
| --- | --- | --- | --- | --- | --- | --- | --- | --- | --- | --- | --- |
|  | **N** | **RR (95%CI)** | ***I*^2^ (%)** | ***P ^a^*** | ***P ^b^*** | **N** | **RR (95%CI)** | ***I*^2^ (%)** | ***P ^a^*** | ***P ^b^*** | |
| **All** | 11 | 1.08 (0.90-1.29) | 75.8 | 0.000 |  | 12 | 1.05 (0.85-1.29) | 60.7 | 0.003 |  | |
| **Region** |  |  |  |  | 0.711 |  |  |  |  | 0.815 | |
| Europe | 5 | 1.02 (0.78-1.33) | 69.1 | 0.012 |  | 5 | 1.04 (0.77-1.40) | 68.2 | 0.013 |  | |
| North America | 6 | 1.12 (0.85-1.48) | 81.8 | 0.000 |  | 7 | 1.05 (0.76-1.45) | 60.7 | 0.018 |  | |
| **Gender** |  |  |  |  | 0.202 |  |  |  |  | 0.699 | |
| Female | 3 | 1.00 (0.76-1.32) | 20.0 | 0.286 |  | 4 | 0.97 (0.60-1.58) | 37.4 | 0.188 |  | |
| Male | 1 | 1.04 (0.66-1.64) | - | - |  | 2 | 1.07 (0.90-1.28) | 0.0 | 0.956 |  | |
| Both | 7 | 1.11 (0.87-1.41) | 84.3 | 0.000 |  | 6 | 1.06 (0.72-1.55) | 78.3 | 0.000 |  | |
| **Type of studies** |  |  |  |  | 0.114 |  |  |  |  | 0.449 | |
| Cohort | 9 | 1.00 (0.82-1.23) | 74.7 | 0.000 |  | 11 | 0.99 (0.81-1.20) | 51.1 | 0.025 |  | |
| Case-control | 2 | 1.44 (1.19-1.74) | 0.0 | 0.837 |  | 1 | 1.74 (1.19-2.54) | - | - |  | |
| **Sample size** |  |  |  |  | 0.160 |  |  |  |  | 0.758 | |
| $<$100,000 | 6 | 1.19 (0.98-1.44) | 38.4 | 0.150 |  | 5 | 1.25 (0.94-1.66) | 45.9 | 0.116 |  | |
| $\geq$100,000 | 5 | 1.00 (0.74-1.35) | 86.3 | 0.000 |  | 7 | 0.94 (0.71-1.23) | 61.5 | 0.016 |  | |
| **Adjustment** |  |  |  |  |  |  |  |  |  |  | |
| **BMI** |  |  |  |  | 0.343 |  |  |  |  | 0.693 | |
| Yes | 7 | 1.05 (0.85-1.31) | 72.5 | 0.001 |  | 9 | 0.98 (0.77-1.25) | 59..8 | 0.011 |  | |
| No | 4 | 1.11 (0.77-1.59) | 81.1 | 0.001 |  | 3 | 1.30 (0.75-2.23) | 66.9 | 0.049 |  | |
| **Alcohol** |  |  |  |  | 0.534 |  |  |  |  | 0.570 | |
| Yes | 5 | 1.05 (0.79-1.38) | 80.4 | 0.000 |  | 5 | 0.92 (0.62-1.34) | 72.4 | 0.006 |  | |
| No | 6 | 1.11 (0.86-1.43) | 72.1 | 0.003 |  | 7 | 1.16 (0.93-1.45) | 41.4 | 0.115 |  | |
| **Vegetable and fruit consumption** |  |  |  |  | 0.463 |  |  |  |  | 0.965 | |
| Yes | 4 | 1.11 (0.81-1.53) | 80.2 | 0.002 |  | 3 | 1.07 (0.61-1.87) | 83.5 | 0.002 |  | |
| No | 7 | 1.05 (0.82-1.35) | 77.0 | 0.000 |  | 9 | 1.05 (0.84-1.31) | 49.2 | 0.046 |  | |
| **Energy intake** |  |  |  |  | 0.342 |  |  |  |  | 0.786 | |
| Yes | 8 | 1.08 (0.85-1.36) | 82.5 | 0.000 |  | 9 | 0.99 (0.74-1.33) | 68.3 | 0.001 |  | |
| No | 3 | 1.07 (0.84-1.35) | 0.0 | 0.511 |  | 3 | 1.12 (0.92-1.36) | 4.6 | 0.350 |  | |

*RR* relative risk, *CI* confidence interval, *BMI* body mass index

^a^ The *P* represents the heterogeneity observed within each subgroup, as determined by the Cochran Q test.

^b^ The *P* is estimated by the meta-regression.

**Table S5. Studies excluded after full-text review and reasons for exclusion**

| **No.** | **First author (Year)** | **Reason for exclusion** |
| --- | --- | --- |
| 1(1) | Burke, Y. D. (1974) | Not relevant exposure |
| 2(2) | Stolzenberg-Solomon, R. Z. (2001) | Not relevant exposure |
| 3(3) | Takeyama, Y. (2005) | Not relevant exposure |
| 4(4) | Jarosz, M. (2007) | Not relevant exposure |
| 5(5) | Zhang, J. J. (2009) | Not relevant exposure |
| 6(6) | Aschebrook-Kilfoy, B. (2011) | Not relevant exposure |
| 7(7) | Johnson, J. (2011) | Not relevant exposure |
| 8(8) | Jansen, R. J. (2012) | Not relevant exposure |
| 9(9) | Lashinger, L. M. (2012) | Not relevant exposure |
| 10(10) | Banim, P. J. (2013) | Not relevant exposure |
| 11(11) | Jansen, R. J. (2013) | Not relevant exposure |
| 12(12) | Matters, G. L. (2014) | Not relevant exposure |
| 13(13) | Casari, I. (2015) | Not relevant exposure |
| 14(14) | Huang, J. Y. (2015) | Not relevant exposure |
| 15(15) | Al-Sabban, A. M. (2016) | Not relevant exposure |
| 16(16) | Arthur, A. E. (2016) | Not relevant exposure |
| 17(17) | Hertzer, K. M. (2016) | Not relevant exposure |
| 18(18) | Lucas, A. L. (2016) | Not relevant exposure |
| 19(19) | Beaney, A. J. (2017) | Not relevant exposure |
| 20(20) | Gordon-Dseagu, V. L. Z. (2017) | Not relevant exposure |
| 21(21) | Zahra, A. (2017) | Not relevant exposure |
| 22(22) | Abdelrehim, M. G. (2018) | Not relevant exposure |
| 23(23) | Asahina, K. (2018) | Not relevant exposure |
| 24(24) | Zheng, J. L. (2018) | Not relevant exposure |
| 25(25) | Zheng, J. L. (2019) | Not relevant exposure |
| 26(26) | Griffin, O. (2020) | Not relevant exposure |
| 27(27) | Iyikesici, M. S. (2020) | Not relevant exposure |
| 28(28) | Kesh, K. (2021) | Not relevant exposure |
| 29(29) | Stolzenberg-Solomon, R. (2021) | Not relevant exposure |
| 30(30) | Xiao, Q. (2021) | Not relevant exposure |
| 31(31) | Zhang, Y. (2021) | Not relevant exposure |
| 32(32) | Zhong, G. C. (2021) | Not relevant exposure |
| 33(33) | Cayssials, V. (2022) | Not relevant exposure |
| 34(34) | Hajihassani, O. (2022) | Not relevant exposure |
| 35(35) | Li, D. (2022) | Not relevant exposure |
| 36(36) | Tayyem, R. (2022) | Not relevant exposure |
| 37(37) | Turati, F. (2022) | Not relevant exposure |
| 38(38) | Yang, L. (2022) | Not relevant exposure |
| 39(39) | Zhang, T. (2022) | Not relevant exposure |
| 40(40) | Afshar, N. (2023) | Not relevant exposure |
| 41(41) | Nikitina, E. (2023) | Not relevant exposure |
| 42(42) | Ghamarzad Shishavan, N. (2024) | Not relevant exposure |
| 43(43) | Hajihassani, O. (2024) | Not relevant exposure |
| 44(44) | Shiraseb F. (2025) | Not relevant exposure |
| 45(45) | Yuan L. (2025) | Not relevant exposure |
| 46(46) | Lin, F (2025) | Not relevant exposure |
| 47(47) | Bastyr-Diego M. (2025) | Not relevant exposure |
| 48(48) | Cobo Diez M.J. (2025) | Not relevant exposure |
| 49(49) | Naudin S. (2025) | Not relevant exposure |
| 50(50) | Shiraseb, F (2025) | Not relevant exposure |
| 51(51) | Katzke V.A. (2025) | Not relevant exposure |
| 52(52) | Cho Y. (2025) | Not relevant exposure |
| 53(53) | Ferrero-Hernández, P (2026) | Not relevant exposure |
| 54(54) | Liu, YQ (2026) | Not relevant exposure |
| 55(55) | Torres-Collado, L (2026) | Not relevant exposure |
| 56(56) | Torres-Collado, Laura (2026) | Not relevant exposure |
| 57(57) | Hadi, M. A. (2016) | Not relevant outcome |
| 58(58) | Ruan, Y. (2019) | Not relevant outcome |
| 59(59) | Rahman, U. U. (2014) | Review or discussion paper |
| 60(60) | Lippi, G. (2016) | Review or discussion paper |
| 61(61) | Miller, P. E. (2016) | Review or discussion paper |
| 62(62) | Zhao, Z. (2017) | Review or discussion paper |
| 63(63) | Han, M. A. (2019) | Review or discussion paper |
| 64(64) | Bulanda, S. (2022) | Review or discussion paper |
| 65(65) | Reng, Q. (2022) | Review or discussion paper |
| 66(66) | Di, Y. (2023) | Review or discussion paper |
| 67(67) | Sanders, L. M. (2023) | Review or discussion paper |
| 68(68) | Sivasubramanian, B. P. (2023) | Review or discussion paper |
| 69(69) | Tsang, M. (2023) | Review or discussion paper |
| 70(70) | Abrignani, Maurizio Giuseppe (2025) | Review or discussion paper |
| 71(71) | Momal, U (2025) | Review or discussion paper |
| 72(72) | Morgan, R. G. (1977) | Conference article or abstract |
| 73(73) | Baghurst, P. A. (1988) | Conference article or abstract |
| 74(74) | Mack, T. (1989) | Conference article or abstract |
| 75(75) | Couper, R. (2002) | Conference article or abstract |
| 76(76) | Hine, R. J. (2003) | Conference article or abstract |
| 77(77) | Culleres, D. B. (2006) | Conference article or abstract |
| 78(78) | Nöthlings (2006) | Conference article or abstract |
| 79(79) | Stolzenberg-Solomon, R. Z (2006) | Conference article or abstract |
| 80(80) | Anderson, K. (2009) | Conference article or abstract |
| 81(81) | Anderson, K. E. (2011) | Conference article or abstract |
| 82(82) | Lukic, S. (2013) | Conference article or abstract |
| 83(83) | Appelhans, K. (2015) | Conference article or abstract |
| 84(84) | Beaney, A. (2015) | Conference article or abstract |
| 85(85) | Moy, K. A. (2015) | Conference article or abstract |
| 86(86) | Norat, T. (2015) | Conference article or abstract |
| 87(87) | Schuz, J. (2015) | Conference article or abstract |
| 88(88) | Arriaga, M. E. (2017) | Conference article or abstract |
| 89(89) | Torramade, E. (2017) | Conference article or abstract |
| 90(90) | Ettrich, T. J. (2018) | Conference article or abstract |
| 91(91) | Pourshams, A. (2018) | Conference article or abstract |
| 92(92) | Porta, C. (2020) | Conference article or abstract |
| 93(93) | Setiawan, V. W. (2020) | Conference article or abstract |
| 94(94) | Ergözen, S. (2021) | Conference article or abstract |
| 95(95) | Malcomson, F. (2023) | Conference article or abstract |
| 96(96) | Ross, M. D. (2023) | Conference article or abstract |
| 97(97) | Vudatha, V. (2023) | Conference article or abstract |
| 98(98) | Nöthlings, U. (2005) | Duplicate study or cohort |
| 99(99) | Cross, A. J. (2007) | Duplicate study or cohort |
| 100(100) | Stolzenberg-Solomon, R. Z. (2007) | Duplicate study or cohort |
| 101(101) | Hu, J. F. (2011) | Duplicate study or cohort |
| 102(102) | Jiao, L. (2015) | Duplicate study or cohort |
| 103(103) | Huang, B. Z. (2019) | Duplicate study or cohort |
| 104(104) | Huang, Y. (2021) | Duplicate study or cohort |
| 105(105) | Raymond, L. (1987) | Inability to provide the required data |
| 106(106) | Voirol, M. (1987) | Inability to provide the required data |
| 107(107) | Zheng, W. (1993) | Inability to provide the required data |
| 108(108) | Anonymous (2004) | Inability to provide the required data |
| 109(109) | Mignone, L. I. (2005) | Inability to provide the required data |
| 110(110) | Artru, P. (2006) | Inability to provide the required data |
| 111(111) | Sun, L. H. (2011) | Inability to provide the required data |
| 112(112) | Tahami, A. N. (2014) | Inability to provide the required data |
| 113(113) | Christensen, L. (2023) | Inability to provide the required data |
| 114(114) | Malcomson, F. C. (2024) | Inability to provide the required data |
| 115(115) | Whyand, M. (2024) | Inability to provide the required data |


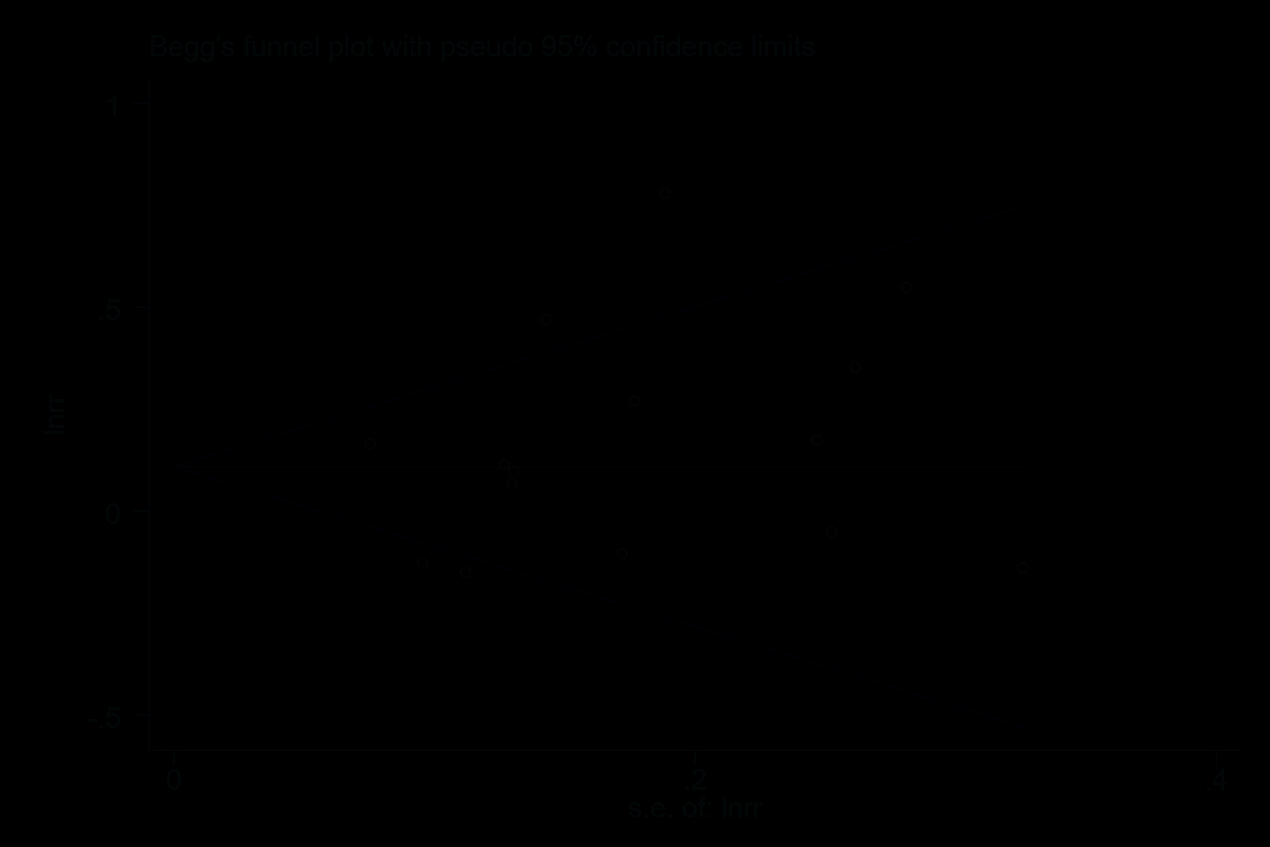


**Figure S1. Funnel plot of pancreatic cancer with high versus low red meat consumption.**


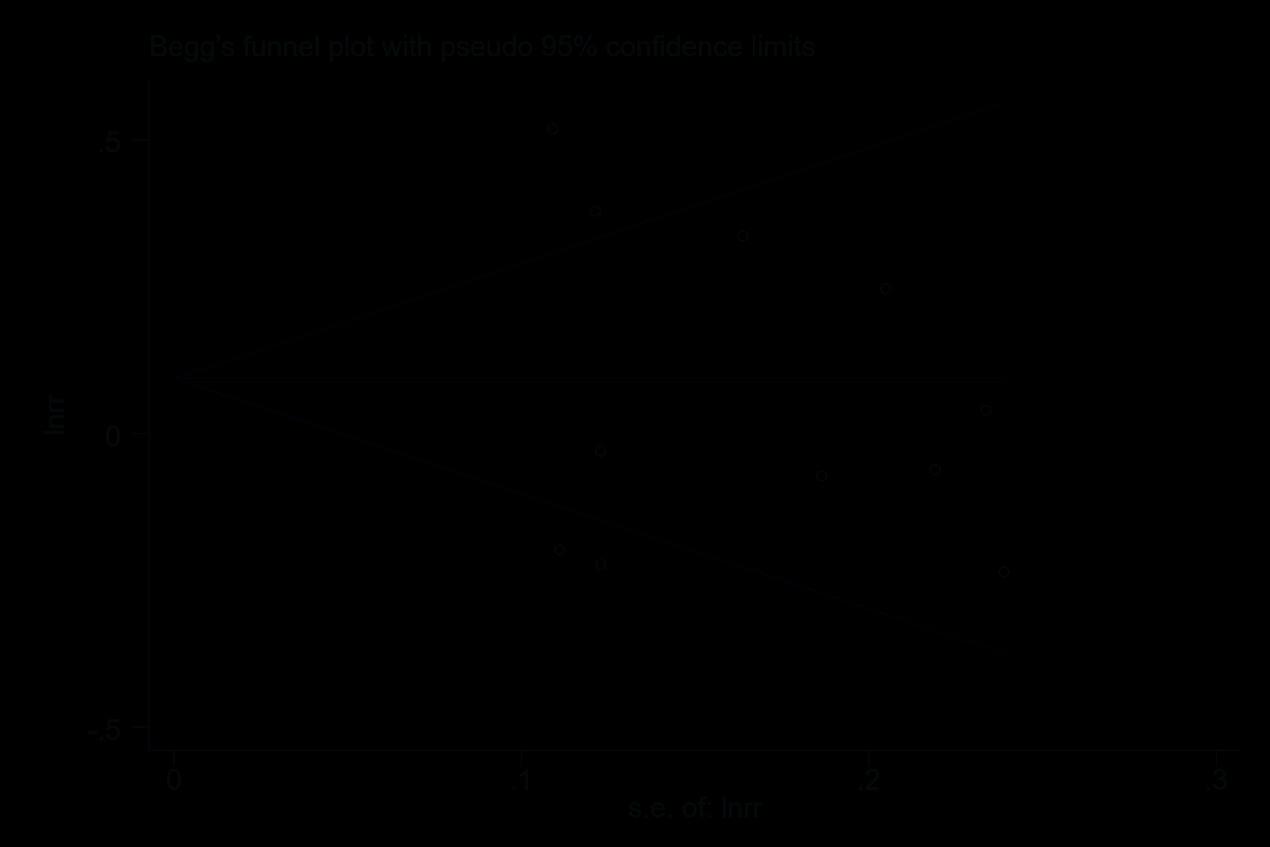


**Figure S2. Funnel plot of pancreatic cancer with high versus low processed meat consumption.**


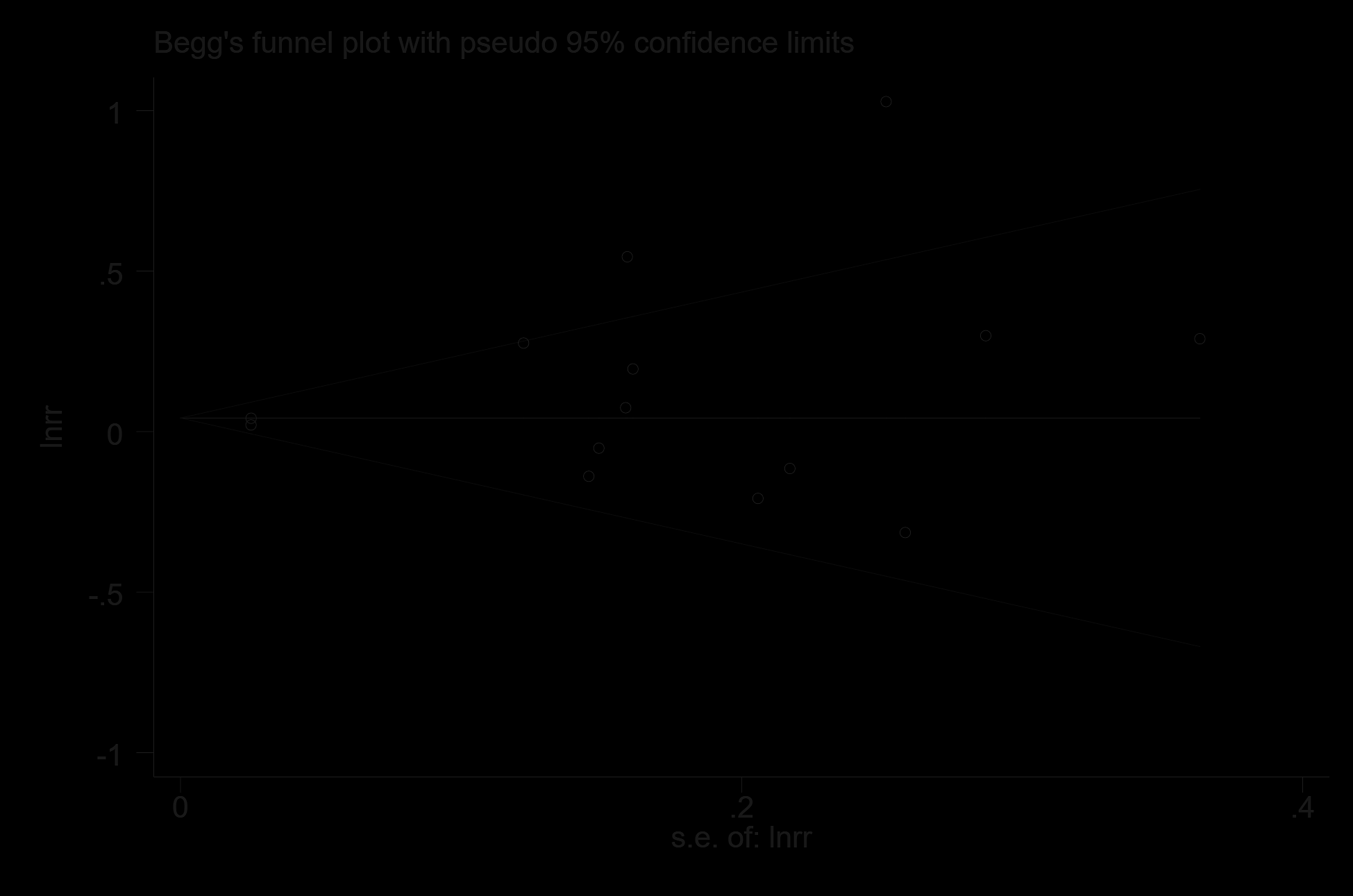


**Figure S3. Funnel plot of pancreatic cancer with per 100g/d increment red meat consumption.**


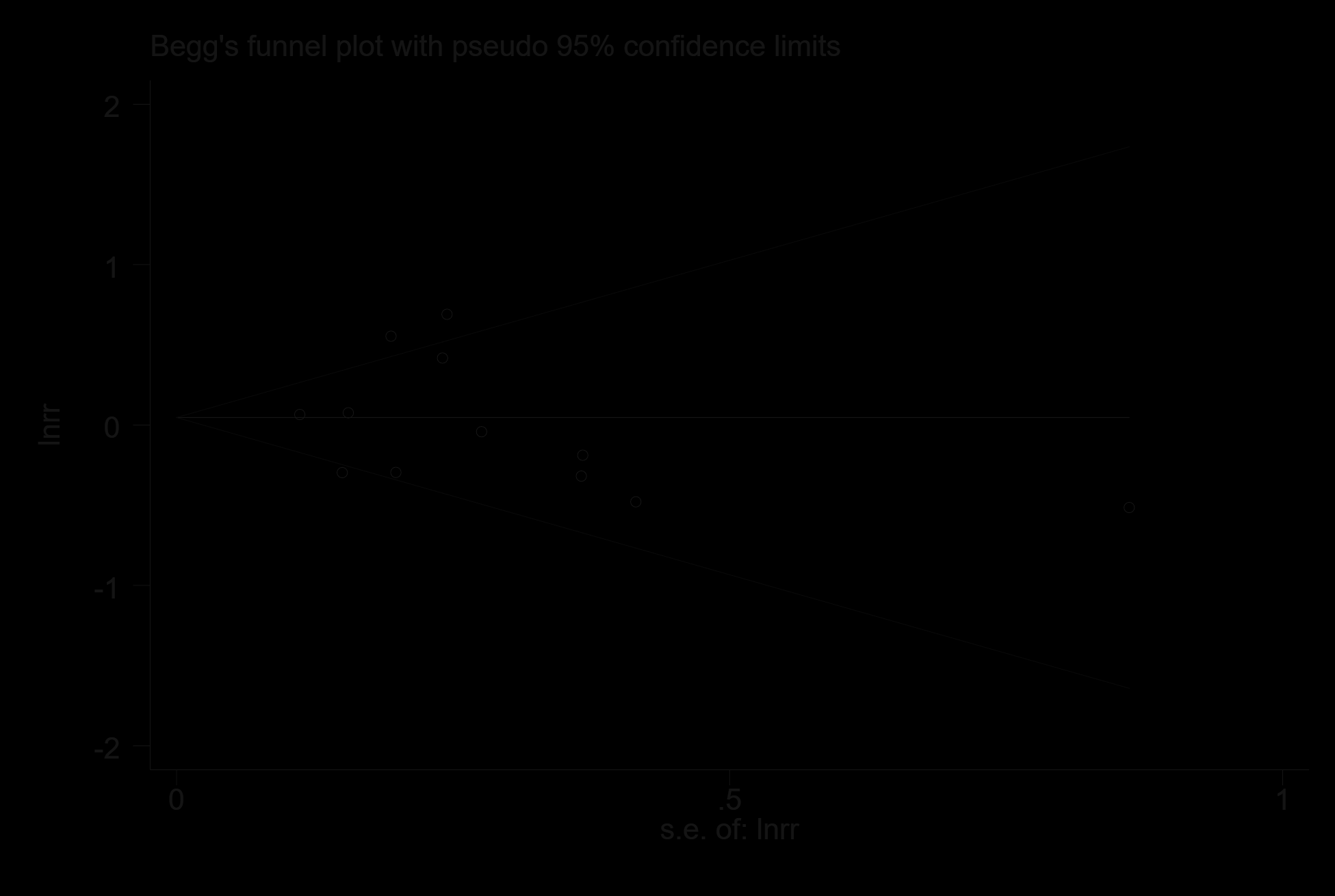


**Figure S4. Funnel plot of pancreatic cancer with per 50g/d increment processed meat consumption.**

**Figure S5. Sensitivity analysis for pancreatic cancer with high versus low red meat consumption.**

 **Figure S6. Sensitivity analysis for pancreatic cancer with high versus low processed meat consumption.**


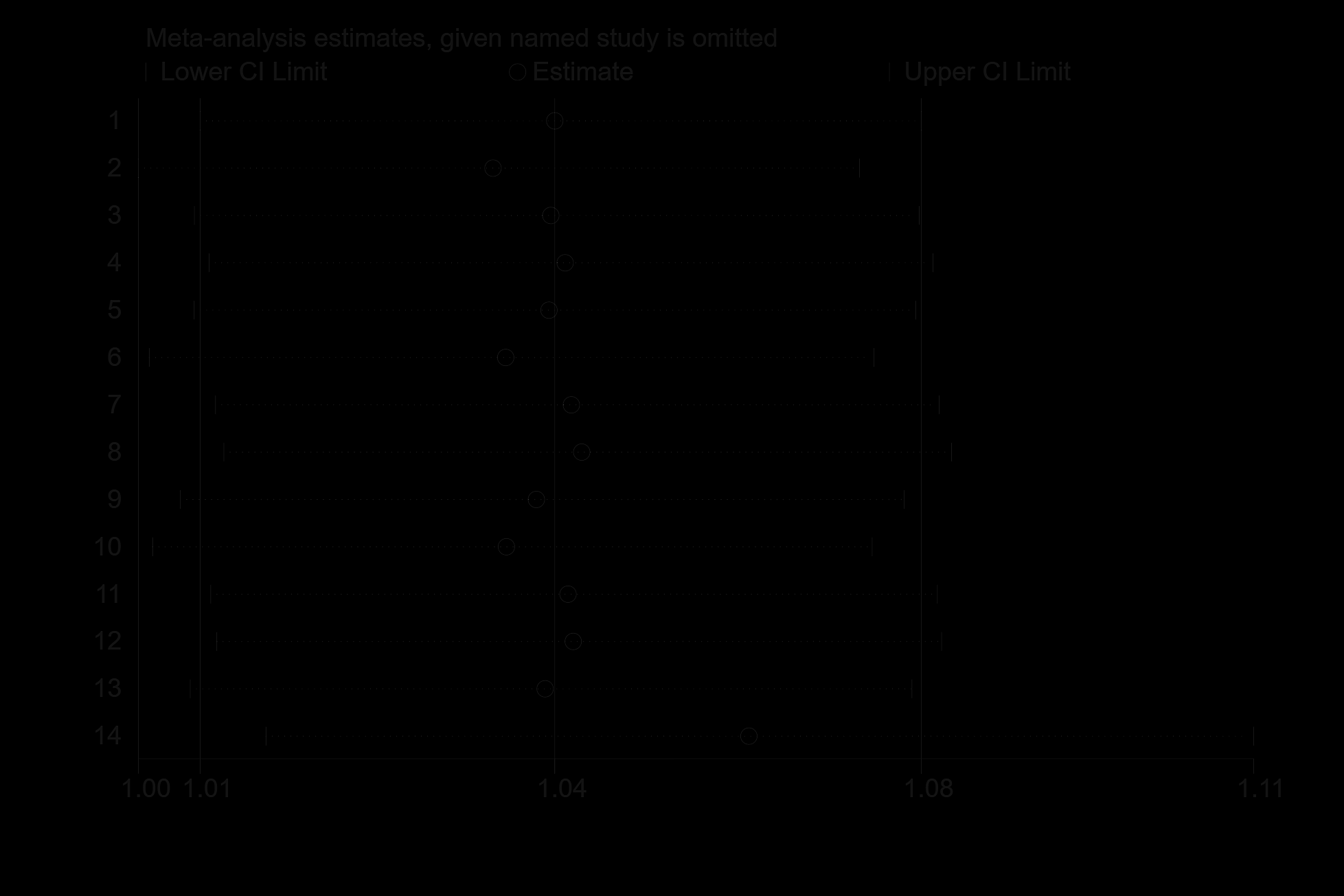
 **Figure S7. Sensitivity analysis for pancreatic cancer with per 100g/d increment red meat consumption.**


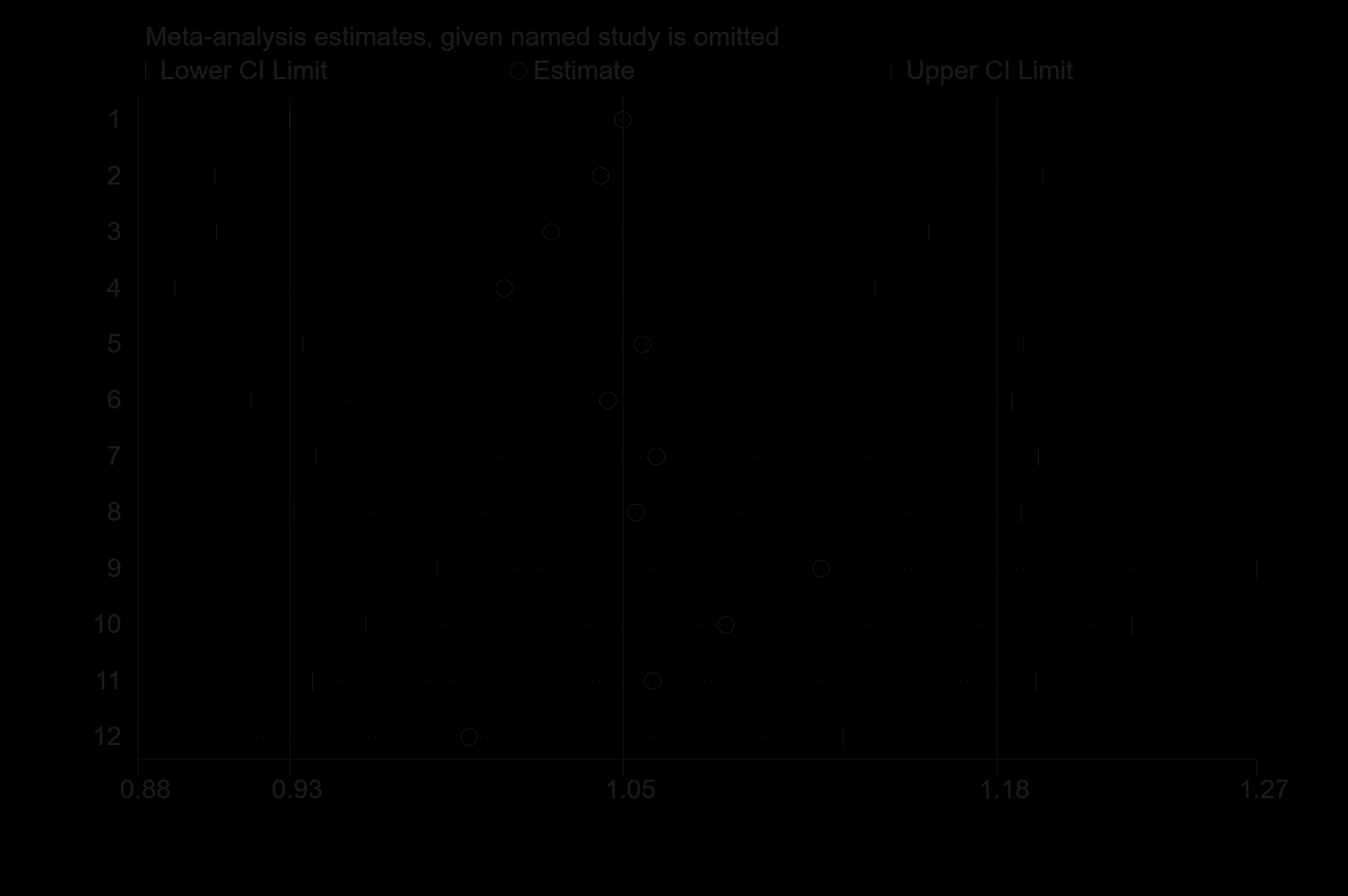


**Figure S8. Sensitivity analysis for pancreatic cancer with per 50g/d increment processed meat consumption.**

1. Burke YD, Stark MJ, Roach SL, Sen SE, Crowell PL. Inhibition of Pancreatic Cancer Growth by the Dietary Isoprenoids Farnesol and Geraniol. *Lipids* (1997) 32(2):151-6. doi: 10.1007/s11745-997-0019-y.

2. Stolzenberg-Solomon RZ, Pietinen P, Barrett MJ, Taylor PR, Virtamo J, Albanes D. Dietary and Other Methyl-Group Availability Factors and Pancreatic Cancer Risk in a Cohort of Male Smokers. *Am J Epidemiol* (2001) 153(7):680-7. doi: 10.1093/aje/153.7.680.

3. Takeyama Y. Dietary Intake as a Risk Factor for Pancreatic Cancer in Japan: High Cholesterol and Low Vitamin C Diet. *J Gastroenterol* (2005) 40(3):324-5. doi: 10.1007/s00535-005-1565-4.

4. Jarosz M, Sekula W, Figurska K, Rychlik E. Pancreatic Cancer and Tobacco Smoking, Diet, and Chronic Pancreatitis in Poland, in 1960-2004. *Gastroenterologia Polska* (2007) 14(5):339

EP - 45.

5. Zhang JJ, Dhakal IB, Gross MD, Lang NP, Kadlubar FF, Harnack LJ, et al. Physical Activity, Diet, and Pancreatic Cancer: A Population-Based, Case-Control Study in Minnesota. *Nutrition and Cancer-an International Journal* (2009) 61(4):457-65. doi: 10.1080/01635580902718941.

6. Aschebrook-Kilfoy B, Cross AJ, Stolzenberg-Solomon RZ, Schatzkin A, Hollenbeck AR, Sinha R, et al. Pancreatic Cancer and Exposure to Dietary Nitrate and Nitrite in the Nih-Aarp Diet and Health Study. *Am J Epidemiol* (2011) 174(3):305-15. Epub 20110617. doi: 10.1093/aje/kwr092.

7. Johnson J, de Mejia EG. Dietary Factors and Pancreatic Cancer: The Role of Food Bioactive Compounds. *Mol Nutr Food Res* (2011) 55(1):58-73. Epub 20101123. doi: 10.1002/mnfr.201000420.

8. Jansen RJ, Robinson DP, Stolzenberg-Solomon RZ, Bamlet WR, Tan XL, Cunningham JM, et al. Polymorphisms in Metabolism Genes May Mediate the Association of Dietary Intake on Pancreatic Cancer Risk. *American Journal of Epidemiology* (2012) 175(SUPPL. 11):S113. doi: <https://dx.doi.org/10.1093/aje/kws258>.

9. Lashinger LM, Harrison LM, Rasmussen AJ, Hursting SD. Dietary Energy Balance Impacts Spontaneous Development of Pancreatic Ductal Adenocarcinoma in the Krasg12d/Ink4a Transgenic Model of Pancreatic Cancer. *Cancer Research* (2012) 72(8 SUPPL. 1). doi: <https://dx.doi.org/10.1158/1538-7445.AM2012-592>.

10. Banim PJ, Luben R, McTaggart A, Welch A, Wareham N, Khaw KT, et al. Dietary Antioxidants and the Aetiology of Pancreatic Cancer: A Cohort Study Using Data from Food Diaries and Biomarkers. *Gut* (2013) 62(10):1489-96. Epub 20120723. doi: 10.1136/gutjnl-2011-301908.

11. Jansen RJ, Robinson DP, Frank RD, Stolzenberg-Solomon RZ, Bamlet WR, Oberg AL, et al. Meat-Related Mutagens and Pancreatic Cancer: Null Results from a Clinic-Based Case-Control Study. *Cancer Epidemiol Biomarkers Prev* (2013) 22(7):1336-9. Epub 20130430. doi: 10.1158/1055-9965.Epi-13-0343.

12. Matters GL, Cooper TK, McGovern CO, Gilius EL, Liao JG, Barth BM, et al. Cholecystokinin Mediates Progression and Metastasis of Pancreatic Cancer Associated with Dietary Fat. *Digestive Diseases and Sciences* (2014) 59(6):1180-91. doi: 10.1007/s10620-014-3201-8.

13. Casari I, Falasca M. Diet and Pancreatic Cancer Prevention. *Cancers (Basel)* (2015) 7(4):2309-17. Epub 20151123. doi: 10.3390/cancers7040892.

14. Huang JY, Butler LM, Wang R, Jin AZ, Koh WP, Yuan JM. Dietary Intake of Vitamin B6 and Choline Are Inversely Associated with Pancreatic Cancer Risk: The Singapore Chinese Health Study. *Cancer Research* (2015) 75(15 SUPPL. 1). doi: <https://dx.doi.org/10.1158/1538-7445.AM2015-1882>.

15. Al-Sabban AM, Nadella S, Burks J, Tucker RD, Smith JP. Cck-Receptor Antagonist Reverses Dietary Fat-Stimulated Growth of Pancreatic Cancer by Decreasing Tumor-Associated Fibrosis. *Gastroenterology* (2016) 150(4 SUPPL. 1):S221

EP - S2.

16. Arthur AE, Delk A, Demark-Wahnefried W, Christein JD, Contreras C, Posey JA, 3rd, et al. Pancreatic Cancer Survivors' Preferences, Barriers, and Facilitators Related to Physical Activity and Diet Interventions. *J Cancer Surviv* (2016) 10(6):981-9. Epub 20160430. doi: 10.1007/s11764-016-0544-5.

17. Hertzer KM, Xu M, Moro A, Dawson DW, Du L, Li G, et al. Robust Early Inflammation of the Peripancreatic Visceral Adipose Tissue during Diet-Induced Obesity in the Krasg12d Model of Pancreatic Cancer. *Pancreas* (2016) 45(3):458-65. doi: 10.1097/mpa.0000000000000497.

18. Lucas AL, Bosetti C, Boffetta P, Negri E, Tavani A, Serafini M, et al. Dietary Total Antioxidant Capacity and Pancreatic Cancer Risk: An Italian Case-Control Study. *Br J Cancer* (2016) 115(1):102-7. Epub 20160512. doi: 10.1038/bjc.2016.114.

19. Beaney AJ, Banim PJR, Luben R, Lentjes MAH, Khaw KT, Hart AR. Higher Meat Intake Is Positively Associated with Higher Risk of Developing Pancreatic Cancer in an Age-Dependent Manner and Are Modified by Plasma Antioxidants <I>a Prospective Cohort Study</I> (<I>Epic</I>-<I>Norfolk</I>) <I>Using Data from Food Diaries</I>. *Pancreas* (2017) 46(5):672-8. doi: 10.1097/mpa.0000000000000819.

20. Gordon-Dseagu VLZ, Thompson FE, Subar AF, Ruder EH, Thiébaut ACM, Potischman N, et al. A Cohort Study of Adolescent and Midlife Diet and Pancreatic Cancer Risk in the Nih-Aarp Diet and Health Study. *Am J Epidemiol* (2017) 186(3):305-17. doi: 10.1093/aje/kwx036.

21. Zahra A, Fath MA, Opat E, Mapuskar KA, Bhatia SK, Ma DC, et al. Consuming a Ketogenic Diet While Receiving Radiation and Chemotherapy for Locally Advanced Lung Cancer and Pancreatic Cancer: The University of Iowa Experience of Two Phase 1 Clinical Trials. *Radiation Research* (2017) 187(6):743

EP - 54. doi: <https://dx.doi.org/10.1667/RR14668.1>.

22. Abdelrehim MG, Mahfouz EM, Ewis AA, Seedhom AE, Afifi HM, Shebl FM. Dietary Factors Associated with Pancreatic Cancer Risk in Minia, Egypt: Principal Component Analysis. *Asian Pac J Cancer Prev* (2018) 19(2):449-55. Epub 20180226. doi: 10.22034/apjcp.2018.19.2.449.

23. Asahina K. Promotion of Pancreatic Cancer Development by Alcohol and Western Diet Feeding. *Alcoholism-Clinical and Experimental Research* (2018) 42:139A-A.

24. Zheng JL, Merchant AT, Wirth MD, Zhang JJ, Antwi SO, Shoaibi A, et al. Inflammatory Potential of Diet and Risk of Pancreatic Cancer in the Prostate, Lung, Colorectal and Ovarian (Plco) Cancer Screening Trial. *International Journal of Cancer* (2018) 142(12):2461-70. doi: 10.1002/ijc.31271.

25. Zheng JL, Wirth MD, Merchant AT, Zhang JJ, Shivappa N, Stolzenberg-Solomon RZ, et al. Inflammatory Potential of Diet, Inflammation-Related Lifestyle Factors, and Risk of Pancreatic Cancer: Results from the Nih-Aarp Diet and Health Study. *Cancer Epidemiology Biomarkers & Prevention* (2019) 28(7):1266-70. doi: 10.1158/1055-9965.Epi-19-0250.

26. Griffin O, Duggan S, Fennelly D, McDermott R, Geoghegan J, Conlon K. Exploring the Feasibility of a Supportive Care Intervention for Patients Undergoing Neo-Adjuvant Chemotherapy for Pancreatic Cancer: The Feed Study (a Fish Oil Supplement, Pancreatic Enzyme Supplement, Exercise Advice and Individualised Dietary Counselling). *HPB* (2020) 22(Supplement 2):S329

EP - S30. doi: <https://dx.doi.org/10.1016/j.hpb.2020.04.315>.

27. Iyikesici MS. Langzeituberlebens-Outcomes Der Metabolisch Unterstutzten Chemotherapie Mit Gemcitabin Oder Folfirinox in Kombination Mit Ketogener Ernahrung, Hyperthermie Und Hyperbarer Sauerstofftherapie Beim Metastasierenden Pankreaskarzinom, Long-Term Survival Outcomes of Metabolically Supported Chemotherapy with Gemcitabine-Based or Folfirinox Regimen Combined with Ketogenic Diet, Hyperthermia, and Hyperbaric Oxygen Therapy in Metastatic Pancreatic Cancer. *Complementary medicine research* (2020) 27(1):31

EP - 9. doi: <https://dx.doi.org/10.1159/000502135>.

28. Kesh K, Banerjee S, Mendez R, Mateo-Victoriano B, Garrido VT, Durden BC, et al. Diet Induced Hyperlipidemia Confers Resistance to Standard Therapy in Pancreatic Cancer by Selecting for "Tumor Protective" Microbial Metabolites and Treatment Refractory Cells. *Pancreas* (2021) 50(7):1070-.

29. Stolzenberg-Solomon R. Are Prediagnostic Biomarkers of Inflammation and an Empirically Based Proinflammatory Dietary Pattern Associated with Poorer Pancreatic Cancer Survival? *J Natl Cancer Inst* (2021) 113(9):1123-4. doi: 10.1093/jnci/djab043.

30. Xiao Q, Jones RR, James P, Stolzenberg-Solomon RZ. Light at Night and Risk of Pancreatic Cancer in the Nih-Aarp Diet and Health Study. *Cancer Res* (2021) 81(6):1616-22. Epub 20210129. doi: 10.1158/0008-5472.Can-20-2256.

31. Zhang Y, Zhang T, Yang W, Geng X, Li G, Chen H, et al. Beneficial Diets and Pancreatic Cancer: Molecular Mechanisms and Clinical Practice. *Frontiers in Oncology* (2021) 11:630972. doi: <https://dx.doi.org/10.3389/fonc.2021.630972>.

32. Zhong GC, Li QJ, Yang PF, Wang YB, Hao FB, Wang K, et al. Low-Carbohydrate Diets and the Risk of Pancreatic Cancer: A Large Prospective Cohort Study. *Carcinogenesis* (2021) 42(5):724-32. doi: 10.1093/carcin/bgab006.

33. Cayssials V, Buckland G, Crous-Bou M, Bonet C, Weiderpass E, Skie G, et al. Inflammatory Potential of Diet and Pancreatic Cancer Risk in the Epic Study. *Eur J Nutr* (2022) 61(5):2313-20. Epub 20220129. doi: 10.1007/s00394-022-02809-y.

34. Hajihassani O, Vaziri-Gohar A, Zarei M, Hue J, Cheng H, Mudigonda A, et al. Understanding the Effects of a Ketogenic Diet against Pancreatic Cancer. *Cancer Research* (2022) 82(12 Supplement). doi: <https://dx.doi.org/10.1158/1538-7445.AM2022-3021>.

35. Li D, Zheng J, Hatia R, Hassan M, Daniel CR. Dietary Intake of Fatty Acids and Risk of Pancreatic Cancer: A Case-Control Study. *J Nutr* (2022) 152(2):439-47. doi: 10.1093/jn/nxab372.

36. Tayyem R, Hammad S, Allehdan S, Al-Jaberi T, Hushki A, Rayyan Y, et al. Dietary Patterns Associated with the Risk of Pancreatic Cancer: Case-Control Study Findings. *Medicine (Baltimore)* (2022) 101(48):e31886. doi: 10.1097/md.0000000000031886.

37. Turati F, Rossi M, Mattioli V, Bravi F, Negri E, La Vecchia C. Diabetes Risk Reduction Diet and the Risk of Pancreatic Cancer. *Eur J Nutr* (2022) 61(1):309-16. Epub 20210802. doi: 10.1007/s00394-021-02646-5.

38. Yang L, TeSlaa T, Ng S, Nofal M, Wang L, Lan T, et al. Ketogenic Diet and Chemotherapy Combine to Disrupt Pancreatic Cancer Metabolism and Growth. *Med* (2022) 3(2):119-36. doi: 10.1016/j.medj.2021.12.008.

39. Zhang T, Wu SS, Xu FY, Chang JP, Guo YX, Zhou ZT, et al. The Association between Dietary Protein Intake and the Risk of Pancreatic Cancer: Evidence from 14 Publications. *Nutrition and Cancer-an International Journal* (2022) 74(9):3172-8. doi: 10.1080/01635581.2022.2059529.

40. Afshar N, Hodge AM, Shivappa N, Hébert JR, Giles GG, English DR, et al. Dietary Inflammatory Index, Alternative Healthy Eating Index-2010, Mediterranean Diet Score and the Risk of Pancreatic Cancer. *Cancer Epidemiol* (2023) 82:102295. Epub 20221114. doi: 10.1016/j.canep.2022.102295.

41. Nikitina E, Alikhanyan K, Neßling M, Richter K, Kaden S, Ernst C, et al. Structural Expression of Bovine Milk and Meat Factors in Tissues of Colorectal, Lung and Pancreatic Cancer Patients. *Int J Cancer* (2023) 153(1):173-82. Epub 20221208. doi: 10.1002/ijc.34374.

42. Ghamarzad Shishavan N, Masoudi S, Mohamadkhani A, Sepanlou SG, Poustchi H, Hekmatdoost A, et al. The Association of Dietary Intake and Plasma Fatty Acid Panel in Pancreatic Cancer Patients: Results from Golestan Cohort Study. *Nutr Health* (2024) 30(2):319-30. Epub 20220721. doi: 10.1177/02601060221114712.

43. Hajihassani O, Zarei M, Tahan S, Gallagher P, Beegan W, Speers J, et al. Targeting Metabolic Vulnerabilities in Pancreatic Cancer: The Synergistic Anti- Tumor Effects of a Ketogenic Diet and Idh1 Inhibition. *Cancer Research* (2024) 84(17). doi: 10.1158/1538-7445.Pancreatic24-b004.

44. F. S, A. K, H. P, A. P, A. E, F. K, et al. Dietary Intake of Animal and Plant Proteins and Risk of Gastrointestinal Cancer Mortality: Results from the Golestan Cohort Study. *Journal of Nutrition* (2025) 155(10):3479-95. doi: 10.1016/j.tjnut.2025.07.026.

45. L. Y, Z. L, J. Z, Y. C, Y. Z, N. W, et al. Combined Impact of Multiple Healthy Lifestyles on Digestive Diseases: A Large Population-Based Prospective Cohort Study. *BMC Medicine* (2025) 23(1):648. doi: 10.1186/s12916-025-04485-1.

46. Lin F, Hu W, Yang C, Cheng B, Chen H, Li J, et al. Associations of Combined Lifestyle and Metabolic Risks with Cancer Incidence in the Uk Biobank Study. *BMC CANCER* (2025) 25(1). doi: 10.1186/s12885-025-13955-x.

47. M. B-D, M. B-L, M. O, A. G, M. B-R, M.A. M-G, et al. Association between an Active Lifestyle and Reduced Incidence of Obesity-Related Cancers in the Seguimiento Universidad De Navarra Cohort. *Preventive Medicine* (2025) 199((Bastyr-Diego, Barberia-Latasa, Olmedo, Gea, Bes-Rastrollo, Martinez-Gonzalez, Toledo) University of Navarra, Department of Preventive Medicine and Public Health, Pamplona, Spain):108386. doi: 10.1016/j.ypmed.2025.108386.

48. M.J. CD, Z. V-R, I. H, J.M. M-M, I.G. G, M.A. M-G, et al. Degree of Food Processing and Incidence of Obesity-Related Cancers in the "Seguimiento Universidad De Navarra" Project. *Journal of Nutrition* (2025) 155(11):3987-96. doi: 10.1016/j.tjnut.2025.08.031.

49. S. N, M. W, N. D, E. E, J. G, H.-O. A, et al. Alcohol Intake and Pancreatic Cancer Risk: An Analysis from 30 Prospective Studies across Asia, Australia, Europe, and North America. *PLOS Medicine* (2025) 22(5):e1004590. doi: 10.1371/journal.pmed.1004590.

50. Shiraseb F, Keshtkar A, Poustchi H, Pourshams A, Etemadi A, Kamangar F, et al. Dietary Intake of Animal and Plant Proteins and Risk of Gastrointestinal Cancer Mortality: Results from the Golestan Cohort Study. *JOURNAL OF NUTRITION* (2025) 155(10):3479-95. doi: 10.1016/j.tjnut.2025.07.026.

51. V.A. K, S. D, A. R, L. A, G. C, G. P, et al. Sex Disparities and Female Reproductive and Hormonal Factors Associated with Risk of Pancreatic Cancer in the European Prospective Investigation into Cancer and Nutrition (Epic) Cohort. *Cancers* (2025) 17(14):2275. doi: 10.3390/cancers17142275.

52. Y. C, J. L, M. G, Y. K, S. J, J. K. A Higher Intake of White-Edible-Colored Fruits and Vegetables Is Associated with Lower Gastrointestinal Cancer Risk among Korean Adults in a Prospective Cohort Study. *Nutrition Research* (2025) 142((Cho, Lee, Gunathilake, Kim) Department of Cancer Biomedical Science, National Cancer Center Graduate School of Cancer Science and Policy, Gyeonggi-do, Goyang-si, South Korea):1-15. doi: 10.1016/j.nutres.2025.08.005.

53. Ferrero-Hernández P, Farías-Valenzuela C, Rezende L, Nilson E, Wahrhaftig J, de Campos L, et al. Cancer Deaths Attributable to Lifestyle Risk Factors in Five Latin American Countries. *PUBLIC HEALTH* (2026) 250. doi: 10.1016/j.puhe.2025.106079.

54. Liu Y, Ma H, Lu Y, Jiang D, Liu L, Zhu Y, et al. Sweet Foods Intake and Risk of Pancreatic Diseases: A Large Cohort Study from the Uk Biobank. *NUTRITION* (2026) 143. doi: 10.1016/j.nut.2025.113032.

55. Torres-Collado L, González-Palacios S, Compañ-Gabucio L, Ojeda-Belokon C, Belisario-Ubeto M, García-de-la-Hera M, et al. Ultra-Processed Food Consumption and Risk of Oesophagus, Stomach, and Pancreatic Cancers: A Multi Case-Control Study. *FRONTIERS IN NUTRITION* (2026) 13. doi: 10.3389/fnut.2026.1764868.

56. Torres-Collado L, González-Palacios S, Compañ-Gabucio LM, Ojeda-Belokon C, Belisario-Ubeto MG, García-de-la-Hera M, et al. Ultra-Processed Food Consumption and Risk of Oesophagus, Stomach, and Pancreatic Cancers: A Multi Case-Control Study. *Frontiers in nutrition* (2026) 13:1764868. doi: 10.3389/fnut.2026.1764868.

57. Hadi MA. Processed Meat as Carcinogen: Time for Health Warning Labels? *Journal of Cancer Policy* (2016) 8:9. doi: <https://dx.doi.org/10.1016/j.jcpo.2016.03.004>.

58. Ruan Y, Poirier AE, Hebert LA, Grevers X, Walter SD, Villeneuve PJ, et al. Estimates of the Current and Future Burden of Cancer Attributable to Red and Processed Meat Consumption in Canada. *Prev Med* (2019) 122:31-9. doi: 10.1016/j.ypmed.2019.03.011.

59. Rahman UU, Sahar A, Khan MI, Nadeem M. Production of Heterocyclic Aromatic Amines in Meat: Chemistry, Health Risks and Inhibition. A Review. *Lwt-Food Science and Technology* (2014) 59(1):229-33. doi: 10.1016/j.lwt.2014.06.005.

60. Lippi G, Mattiuzzi C, Cervellin G. Meat Consumption and Cancer Risk: A Critical Review of Published Meta-Analyses. *Critical Reviews in Oncology/Hematology* (2016) 97:1

EP - 14. doi: <https://dx.doi.org/10.1016/j.critrevonc.2015.11.008>.

61. Miller PE, Alexander D. A Review and Meta-Analysis of Prospective Studies of Red and Processed Meat and Pancreatic Cancer. *Faseb Journal* (2016) 30.

62. Zhao Z, Yin Z, Pu Z, Zhao Q. Association between Consumption of Red and Processed Meat and Pancreatic Cancer Risk: A Systematic Review and Meta-Analysis. *Clin Gastroenterol Hepatol* (2017) 15(4):486-93.e10. Epub 20160928. doi: 10.1016/j.cgh.2016.09.143.

63. Han MA, Zeraatkar D, Guyatt GH, Vernooij RWM, El Dib R, Zhang Y, et al. Reduction of Red and Processed Meat Intake and Cancer Mortality and Incidence a Systematic Review and Meta-Analysis of Cohort Studies. *Annals of Internal Medicine* (2019) 171(10):711

EP - 20. doi: <https://dx.doi.org/10.7326/M19-0699>.

64. Bulanda S, Janoszka B. Consumption of Thermally Processed Meat Containing Carcinogenic Compounds (Polycyclic Aromatic Hydrocarbons and Heterocyclic Aromatic Amines) Versus a Risk of Some Cancers in Humans and the Possibility of Reducing Their Formation by Natural Food Additives-a Literature Review. *International Journal of Environmental Research and Public Health* (2022) 19(8). doi: 10.3390/ijerph19084781.

65. Reng Q, Zhu LL, Feng L, Li YJ, Zhu YX, Wang TT, et al. Dietary Meat Mutagens Intake and Cancer Risk: A Systematic Review and Meta-Analysis. *Frontiers in Nutrition* (2022) 9. doi: 10.3389/fnut.2022.962688.

66. Di Y, Ding L, Gao L, Huang H. Association of Meat Consumption with the Risk of Gastrointestinal Cancers: A Systematic Review and Meta-Analysis. *BMC Cancer* (2023) 23(1):782. Epub 20230823. doi: 10.1186/s12885-023-11218-1.

67. Sanders LM, Wilcox ML, Maki KC. Red Meat Consumption and Risk Factors for Type 2 Diabetes: A Systematic Review and Meta-Analysis of Randomized Controlled Trials. *European Journal of Clinical Nutrition* (2023) 77(2):156-65. doi: 10.1038/s41430-022-01150-1.

68. Sivasubramanian BP, Dave M, Panchal V, Saifa-Bonsu J, Konka S, Noei F, et al. Comprehensive Review of Red Meat Consumption and the Risk of Cancer. *Cureus* (2023) 15(9):e45324. Epub 20230915. doi: 10.7759/cureus.45324.

69. Tsang M, Dong J, DuMontier C, Neuendorff NR, Presley CJ. "Celebrating Resilience": A Review of Abstracts on Functional Resilience at the International Society of Geriatric Oncology 2022 Annual Meeting. *Journal of Geriatric Oncology* (2023) 14(7):101587. doi: <https://dx.doi.org/10.1016/j.jgo.2023.101587>.

70. Abrignani MG, Lucà F, Abrignani V, Nucara M, Grosseto D, Lestuzzi C, et al. Risk Factors and Prevention of Cancer and Cvds: A Chicken and Egg Situation. *Journal of clinical medicine* (2025) 14(9). doi: 10.3390/jcm14093083.

71. Momal U, Naeem H, Aslam F, Shahbaz M, Imran M, Hussain M, et al. Recent Perspectives on Meat Consumption and Cancer Proliferation. *JOURNAL OF FOOD PROCESSING AND PRESERVATION* (2025) 2025(1). doi: 10.1155/jfpp/6567543.

72. Morgan RG, Wormsley KG. Progress Report. Cancer of the Pancreas. *Gut* (1977) 18(7):580-96. doi: 10.1136/gut.18.7.580.

73. Baghurst PA, McMichael AJ, Slavotinek AH. Diet and Cancer of the Pancreas, Gallbladder and Bile-Ducts. *Community Health Studies* (1988) 12(4):463-.

74. Mack T, Boyle P, Pour PM. Summary of the Second Meeting of the International Pancreatic Cancer Study Group (Ipcsg). *International Journal of Pancreatology* (1989) 5(4):387

EP - 9.

75. Couper R, Belli D, Durie P, Gaskin K, Sarles J, Werlin S. Pancreatic Disorders and Cystic Fibrosis: Working Group Report of the First World Congress of Pediatric Gastroenterology, Hepatology, and Nutrition. *Journal of Pediatric Gastroenterology and Nutrition* (2002) 35(SUPPL. 2):S213

EP - S23. doi: <https://dx.doi.org/10.1097/00005176-200208002-00020>.

76. Hine RJ, Srivastava S, Milner JA, Ross SA. Nutritional Links to Plausible Mechanisms Underlying Pancreatic Cancer: A Conference Report. *Pancreas* (2003) 27(4):356-66. doi: 10.1097/00006676-200311000-00014.

77. Culleres DB, Black R, Boesten J, Boobis A, Hardy A, Hart A, et al. Opinion of the Scientific Panel on Plant Health, Plant Protection Products and Their Residues on a Request from Efsa Related to the Evaluation of Dichlorvos in the Context of Council Directive 91/414/Eec. *Efsa Journal* (2006) 4(4). doi: 10.2903/j.efsa.2006.343.

78. Nöthlings. Meat and Fat Intake as Risk Factors for Pancreatic Cancer:: The Multiethnic Cohort Study (Vol 97, Pg 1458, 2005). *Journal of the National Cancer Institute* (2006) 98(11):796-. doi: 10.1093/jnci/djj238.

79. Stolzenberg-Solomon RZ, Cross AJ, Silverman D, Thompson FE, Kipnis V, Subar AF, et al. Meat and Meat Mutagen Intake and Pancreatic Cancer Risk in the Nih-Aarp Diet and Health Study. *American Journal of Epidemiology* (2006) 163(11):S109-S. doi: 10.1093/aje/163.suppl_11.S109-b.

80. Anderson K, Mongin S, Sinha R, Gross M, Church T. Pancreatic Cancer Risk: Associations with Meat-Derived Carcinogen Intake. *Cancer Research* (2009) 69.

81. Anderson KE, Mongin SJ, Sinha R, Stolzenberg-Solomon R, Gross MD, Ziegler RG, et al. Pancreatic Cancer Risk Update: Associations with Meat-Derived Carcinogen Intake in the Prostate, Lung, Colorectal, and Ovarian Cancer Screening Trial (Plco) Cohort. *Cancer Research* (2011) 71. doi: 10.1158/1538-7445.Am2011-1907.

82. Lukic S, Popovic D, Milutinovic AS, Knezevic D, Knezevic S, Milicic B, et al. Characteristics of Patients with Chronic Pancreatitis and Pancreatic Cancer. *Pancreatology* (2013) 13(3 SUPPL. 1):S45.

83. Appelhans K, Frankos V, Najeeullah R, Morgan J. Reports Alleged to Be Associated with an Herbalife Food Product Are Not Properly Evaluated for Alternative Etiologies. *Investigacion Clinica (Venezuela)* (2015) 56(3):336

EP - 8.

84. Beaney A, Banim PJ, Luben R, Khaw KT, Hart AR. Meat and the Risk of Pancreatic Cancer: A Prospective Cohort Study (Epic-Norfolk) Using Data from Food Diaries. *Gut* (2015) 64:A302-A. doi: 10.1136/gutjnl-2015-309861.652.

85. Moy KA, Potischman N, Thompson FE, Subar A, Ruder EH, Thiebaut ACM, et al. Adolescent and Mid-Life Diet: Risk of Pancreatic Cancer in the Nih-Aarp Diet and Health Study. *Cancer Research* (2015) 75. doi: 10.1158/1538-7445.Am2015-1876.

86. Norat T, Scoccianti C, Boutron-Ruault MC, Anderson A, Berrino F, Cecchini M, et al. European Code against Cancer 4th Edition: Diet and Cancer. *Cancer Epidemiol* (2015) 39 Suppl 1:S56-66. Epub 20150709. doi: 10.1016/j.canep.2014.12.016.

87. Schuz J, Espina C, Villain P, Herrero R, Leon ME, Minozzi S, et al. European Code against Cancer 4th Edition: 12 Ways to Reduce Your Cancer Risk. *Cancer Epidemiology* (2015) 39(Supplement1):S1

EP - S10. doi: <https://dx.doi.org/10.1016/j.canep.2015.05.009>.

88. Arriaga ME, Laaksonen MA, Canfell K, Macinnis R, Banks E, Giles G, et al. Population-Level Relevance of Lifestyle-Related Risk Factors for Pancreatic Cancer in Australia. *Cancer Research* (2017) 77(13 Supplement 1). doi: <https://dx.doi.org/10.1158/1538-7445.AM2017-2283>.

89. Torramade E. United European Gastroenterology Week (Uegw) 2017 - 25<Ovid:Sup>Th</Ovid:Sup> Annual Conference (October 28-November 1, 2017 - Barcelona, Spain). *Drugs of Today* (2017) 53(11):619

EP - 23. doi: <https://dx.doi.org/10.1358/dot.2017.53.11.2739611>.

90. Ettrich TJ, Ebert M, Lorenzen S, Moehler M, Vogel A, Witkowski L, et al. Asco- and Esmo-Update 2017 - Highlights of the 53meeting of the American Society of Clinical Oncology/Asco 2017 and European Society for Medical Oncology/Esmo Congress 2017. *Zeitschrift fur Gastroenterologie* (2018) 56(4):384

EP - 97. doi: <https://dx.doi.org/10.1055/s-0044-101757>.

91. Pourshams A. Risk Factors and Epidemiological Features of Pancreatic Cancer in Iran. *Annals of Oncology* (2018) 29(Supplement 5):v41. doi: <https://dx.doi.org/10.1093/annonc/mdy151>.

92. Porta C, Bamias A, Danesh FR, Debska-Slizien A, Gallieni M, Gertz MA, et al. Kdigo Controversies Conference on Onco-Nephrology: Understanding Kidney Impairment and Solid-Organ Malignancies, and Managing Kidney Cancer. *Kidney International* (2020) 98(5):1108

EP - 19. doi: <https://dx.doi.org/10.1016/j.kint.2020.06.046>.

93. Setiawan VW, Wang SR, Stram D, Wu L, Le Marchand L, Shu XO, et al. Red Meat Consumption and Pancreatic Cancer Risk in Two Prospective Studies of Racially Diverse Populations. *Cancer Epidemiology Biomarkers & Prevention* (2020) 29(6). doi: 10.1158/1538-7755.Disp19-c035.

94. Ergözen S, Gümüş T. Long-Term Survival Outcomes of Metabolically Supported Chemotherapy with Gemcitabine-Based or Folfirinox Regimen Combined with Ketogenic Diet, Hyperthermia, and Hyperbaric Oxygen Therapy in Metastatic Pancreatic Cancer: A Letter to the Editor. *Complement Med Res* (2021) 28(3):270-1. Epub 20210218. doi: 10.1159/000514475.

95. Malcomson F, Parra-Soto S, Ho F, Celis-Morales C, Sharp L, Mathers J. Abbreviated Score to Assess Adherence to the 2018 Wcrf/Aicr Cancer Prevention Recommendations and

Risk of Cancer in the Uk Biobank Cohort. *Current Developments in Nutrition* (2023) 7(Supplement 1):100457. doi: <https://dx.doi.org/10.1016/j.cdnut.2023.100457>.

96. Ross MD, Turner JE, Orange ST, Metcalfe RS. Editorial: The Influence of Lifestyle Factors on Cancer Biology and Treatment Efficacy. *Frontiers in Physiology* (2023) 14:1254151. doi: <https://dx.doi.org/10.3389/fphys.2023.1254151>.

97. Vudatha V, Liu C, He J, Freudenberger D, Wages N, Trevino J. Evaluating Relationship between Food Environment and Pancreatic Cancer Demographics and Outcomes. *Cancer Epidemiology Biomarkers & Prevention* (2023) 32(12). doi: 10.1158/1538-7755.Disp23-a102.

98. Nöthlings U, Wilkens LR, Murphy SP, Hankin JH, Henderson BE, Kolonel LN. Meat and Fat Intake as Risk Factors for Pancreatic Cancer: The Multiethnic Cohort Study. *J Natl Cancer Inst* (2005) 97(19):1458-65. doi: 10.1093/jnci/dji292.

99. Cross AJ, Leitzmann MF, Gail MH, Hollenbeck AR, Schatzkin A, Sinha R. A Prospective Study of Red and Processed Meat Intake in Relation to Cancer Risk. *Plos Medicine* (2007) 4(12):1973-84. doi: 10.1371/journal.pmed.0040325.

100. Stolzenberg-Solomon RZ, Cross AJ, Silverman DT, Schairer C, Thompson FE, Kipnis V, et al. Meat and Meat-Mutagen Intake and Pancreatic Cancer Risk in the Nih-Aarp Cohort. *Cancer Epidemiol Biomarkers Prev* (2007) 16(12):2664-75. doi: 10.1158/1055-9965.Epi-07-0378.

101. Hu JF, La Vecchia C, Morrison H, Negri E, Mery L, Canadian Canc Registries E. Salt, Processed Meat and the Risk of Cancer. *European Journal of Cancer Prevention* (2011) 20(2):132-9. doi: 10.1097/CEJ.0b013e3283429e32.

102. Jiao L, Stolzenberg-Solomon R, Zimmerman TP, Duan ZG, Chen L, Kahle L, et al. Dietary Consumption of Advanced Glycation End Products and Pancreatic Cancer in the Prospective Nih-Aarp Diet and Health Study. *American Journal of Clinical Nutrition* (2015) 101(1):126-34. doi: 10.3945/ajcn.114.098061.

103. Huang BZ, Stram DO, Le Marchand L, Haiman CA, Wilkens LR, Pandol SJ, et al. Interethnic Differences in Pancreatic Cancer Incidence and Risk Factors: The Multiethnic Cohort. *Cancer Med* (2019) 8(7):3592-603. Epub 20190508. doi: 10.1002/cam4.2209.

104. Huang Y, Liu F, Chen AM, Yang PF, Peng Y, Gong JP, et al. Type 2 Diabetes Prevention Diet and the Risk of Pancreatic Cancer: A Large Prospective Multicenter Study. *Clin Nutr* (2021) 40(11):5595-604. Epub 20210924. doi: 10.1016/j.clnu.2021.09.037.

105. Raymond L, Infante F, Tuyns AJ. Diet and Cancer of the Pancreas. *Gastroenterologie Clinique et Biologique* (1987) 11(6-7):488

EP - 92.

106. Voirol M, Infante F, Raymond L. Nutrition and Cancer of Pancreas. *Schweizerische Medizinische Wochenschrift* (1987) 117(29):1101

EP - 4.

107. Zheng W, McLaughlin JK, Gridley G, Bjelke E, Schuman LM, Silverman DT, et al. A Cohort Study of Smoking, Alcohol Consumption, and Dietary Factors for Pancreatic Cancer (United States). *Cancer Causes Control* (1993) 4(5):477-82. doi: 10.1007/bf00050867.

108. Anonymous. Dealwatch A1 - Anonymous. *Current Drug Discovery* (2004) (JUNE):13

EP - 4.

109. Mignone LI, Smith-Warner SA. Meat and Fat Intake and Pancreatic Cancer in a Pooled Analysis of Prospective Studies. *Cancer Epidemiology Biomarkers & Prevention* (2005) 14(11):2697S-S.

110. Artru P, Lledo G. Meat Consumption and Cancer Risks: The Pancreas Too! *Acta Endoscopica* (2006) 36(2):205

EP - 6. doi: <https://dx.doi.org/10.1007/bf03006419>.

111. Sun LH. Correlation between Dietary Factors and the Risk of Pancreatic Cancer. *World Chinese Journal of Digestology* (2011) 19(4):410

EP - 5.

112. Tahami AN, Khanjani N, Feyzabadi VY, Varzandeh M, Haghdoost AA. Opium as a Risk Factor for Upper Gastrointestinal Cancers: A Population-Based Case-Control Study in Iran. *Archives of Iranian Medicine* (2014) 17(1):2-6.

113. Christensen L, Calquin M, Daly S. Does the Consumption of Red and Processed Meats Increase the Risk of Cancer? *American Family Physician* (2023) 107(4):424

EP - 5.

114. Malcomson FC, Parra-Soto S, Ho FK, Celis-Morales C, Sharp L, Mathers JC. Abbreviated Score to Assess Adherence to the 2018 Wcrf/Aicr Cancer Prevention Recommendations and Risk of Cancer in the Uk Biobank. *Cancer Epidemiology Biomarkers & Prevention* (2024) 33(1):33-42. doi: 10.1158/1055-9965.Epi-23-0923.

115. Whyand M. The Role of Lifestyle and Diet in Pancreatic Cancer Prevention. *Journal of the Pancreas* (2024) 25(3). doi: 10.35841/1590-8577-25.3.867.
